# Chiral Brønsted Acid Catalyzed Cascade Alcohol Deprotection and Enantioselective Cyclization

**DOI:** 10.1021/acsomega.3c08869

**Published:** 2023-12-29

**Authors:** Joshua A. Frost, Sarah M. Korb, Fiona E. Green, Kala C. Youngblood, Kimberly S. Petersen

**Affiliations:** Department of Chemistry and Biochemistry, University of North Carolina at Greensboro, 320 College Avenue, Greensboro, North Carolina 27412, United States

## Abstract

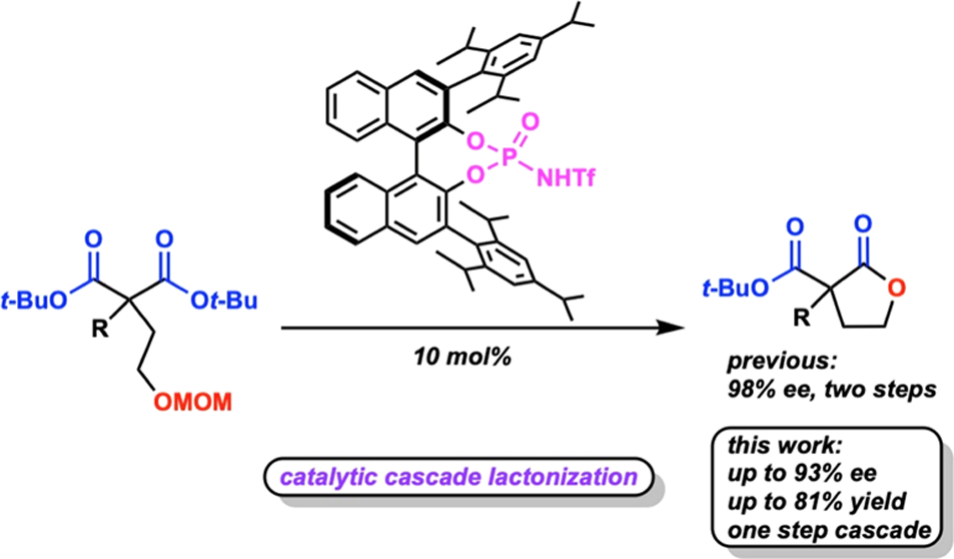

The protection–deprotection sequence is vital to organic synthesis. Here, we describe a novel catalytic cascade where a chiral Brønsted acid selectively removes ether protecting groups and catalyzes intramolecular cyclization in one pot. We tested three model substrates from our previous work and investigated the rate of deprotection through gas chromatography (GC) studies. This work builds on our stereoselective synthesis of lactones by streamlining our synthesis. It also opens the door for additional investigations into other catalytic cascade reactions using chiral Brønsted acid catalysts.

## Introduction

Chemical protecting groups have seen ubiquitous use throughout organic synthesis. Protection and deprotection of alcohol functional groups have been studied extensively, and the advancement of alcohol protection/deprotection sequences has allowed for chemoselective reaction development. Alcohol groups are commonly converted to ethers to mask their reactivity, varying from silyl ether protection to carbon-based ethers like tetrahydropyranyl ethers.^1,2^ While protecting groups allow for better selectivity, their use is not without drawbacks, including the addition of multiple synthetic steps and potential effects on the overall yields of reaction sequences.

Chiral Brønsted acid catalyzed domino lactonizations are known in the literature. Bartoccini and co-workers reported stereoselective formation of benzo and naphthofuranones using chiral phosphoric acid catalysis.^3^ Furthermore, catalytic deprotections are well-known in the chemical literature. Many involve the use of metal- or Lewis acid catalyzed reagents to accomplish this transformation. For example, cerium(III),^4^ cerium(IV),^5^ and bismuth(III)^6^ compounds have been used in the catalytic removal of acetal protecting groups. For the catalytic deprotection of alcohols, specifically, Ce(OTf)_4_^7^ has been used to remove trityl ethers catalytically. Additionally, catalytic removals of silyl ethers to form alcohols have been reported.^8,9^

Reports of Brønsted acid catalyzed deprotections are rarer in the literature. Karimi and Zareyee reported removal of silyl ethers via sulfuric-acid-functionalized nanoporous silica.^10^ Similarly, Iimura et al. reported removal of silyl-protected alcohol groups via polystyrene-supported sulfuric acids.^11^ Despite these, the literature surrounding Brønsted acid catalyzed alcohol deprotections is extremely limited.

**Scheme 1:**
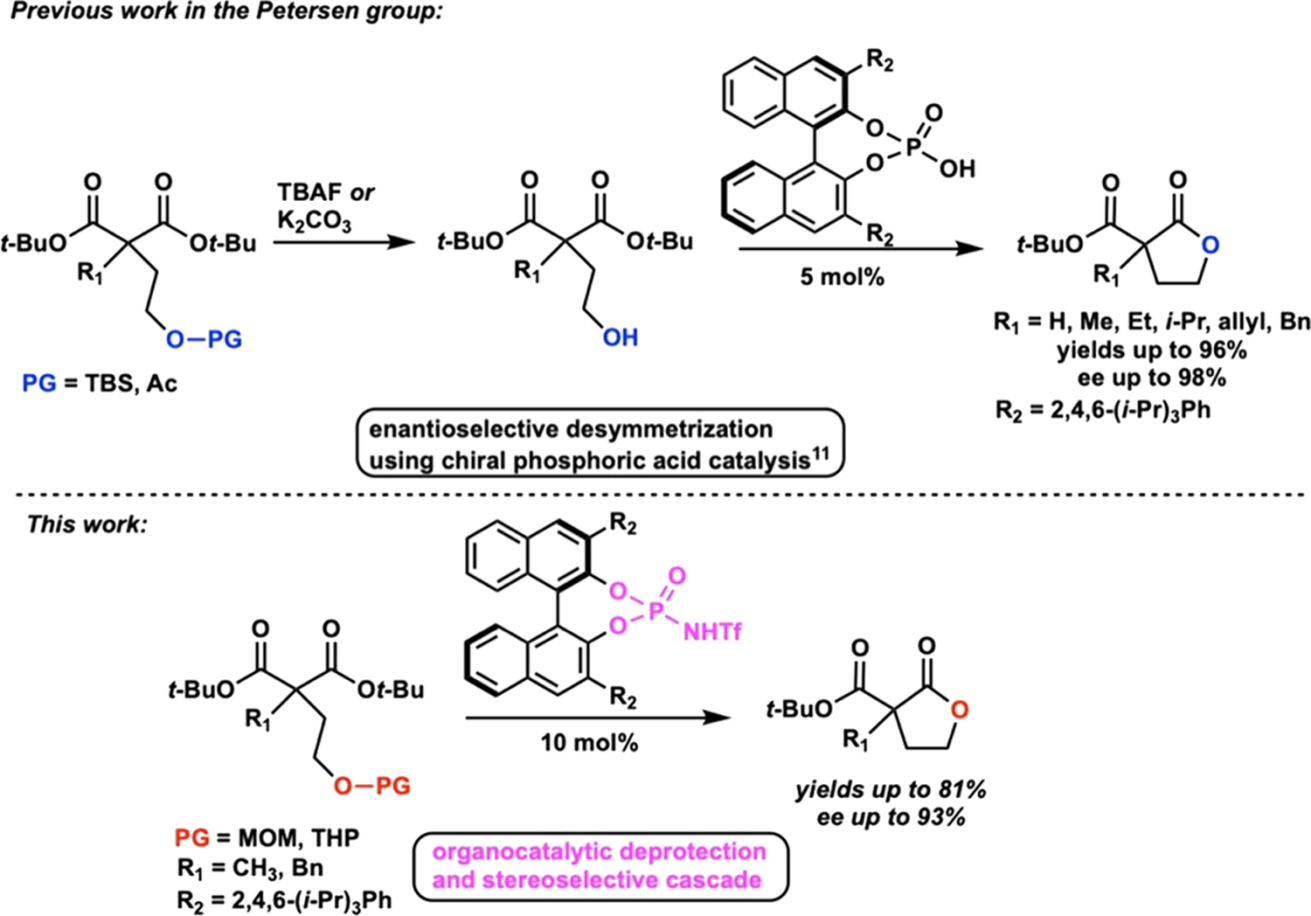
Desymmetrizations of Prochiral Malonates to Lactones

Here, we have developed an organocatalytic deprotection and stereoselective cascade cyclization of ether-protected alcohols to form lactones. To the best of our knowledge, this represents the first use of an organic Brønsted acid to catalyze both alcohol deprotection and stereoselective cyclization in one pot. This reaction methodology builds on the previous Petersen group methodology for the synthesis of lactones (Scheme 1). Whereas the previous Petersen group methodology was limited to a deprotection to a free alcohol followed by diester lactonization, this methodology outlines a reaction that uses a stronger chiral Brønsted acid that allows for a unique cascade transformation from protected alcohol to lactonization to occur. The development of this methodology presents a potential opportunity for the growth of cascade deprotection reactions for further use in organic synthesis.

## Results and Discussion

Our investigation began by envisioning a more elegant reaction pathway than our initial reaction pathway, involving an acid-labile protecting group that is removed in one pot by our chiral acid.

**Scheme 2:**
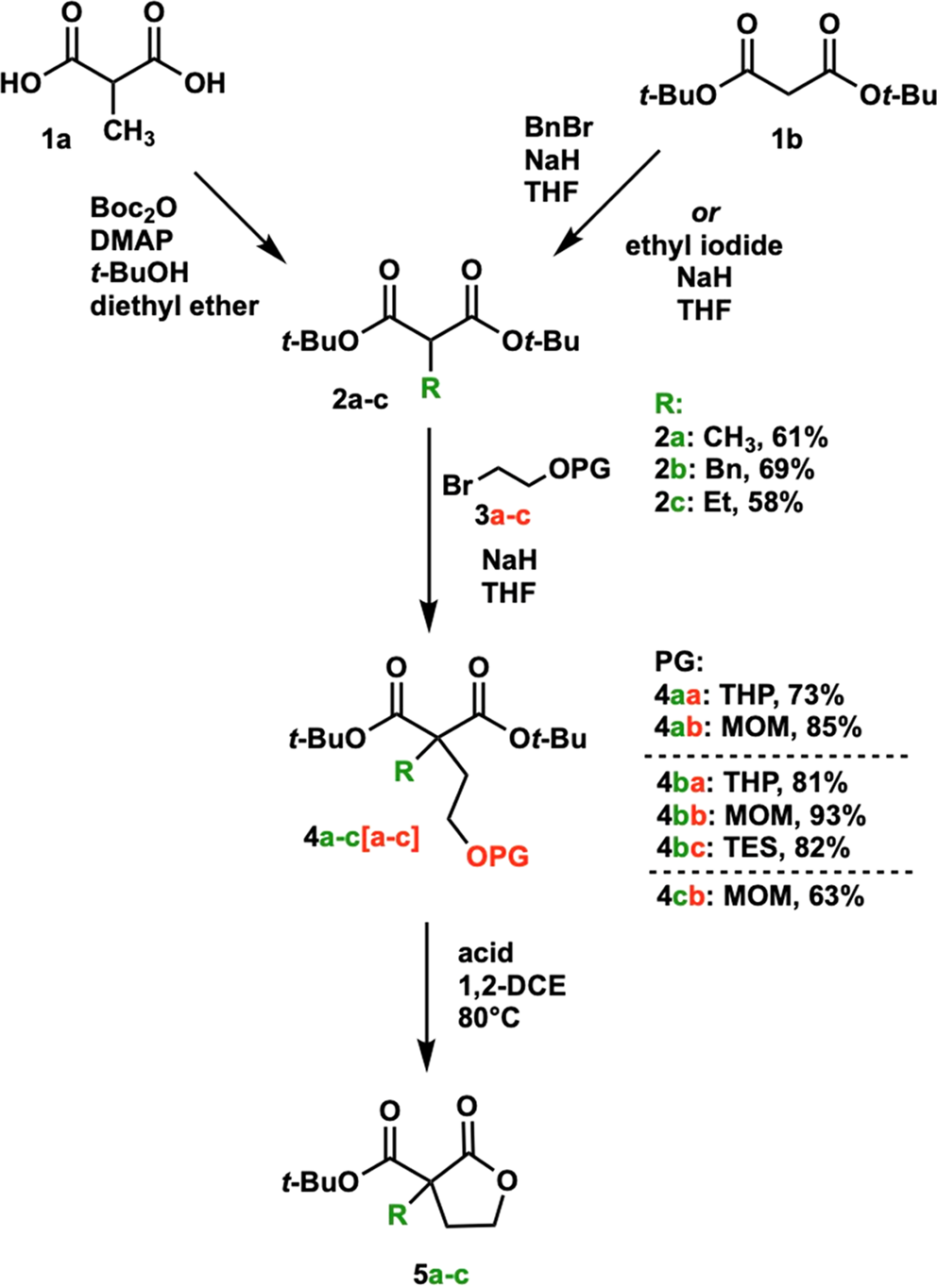
Starting Material Synthesis and Lactone Formation

We initially used α-substituted diesters to test the feasibility of the cascade. The enantioselectivity of these substrates had been established by previously published reactions in the Petersen group.^12^ As such, we chose the methyl-, ethyl-, and benzyl-substituted esters **4a**–**c** as representative examples for a novel cascade reaction. The starting methyl malonate **2a** was synthesized readily from the diacid following our previously published procedure.^13^ The malonate was further alkylated with a protected alcohol **3a**–**b**, giving compounds **4a[*a***–***b*]**. In a similar fashion, malonates **4b**–**c[*a***–***c*]** were synthesized from the starting unsubstituted malonate **1b** following a first alkylation with benzyl bromide or iodoethane to give **2b** and **2c** and a second alkylation with the protected alkylating agent **3a**–**c** (Scheme 2). These tetrahydropyranyl (THP)-, methoxymethyl (MOM)-, and triethylsilyl (TES)-protected alcohols were chosen not only based on their lability toward Brønsted acids but also to evaluate whether such stereoselective deprotection/cyclization cascades are selective only toward ether protecting groups or if silyl groups could be subjected to this cascade.

**Figure 1:**
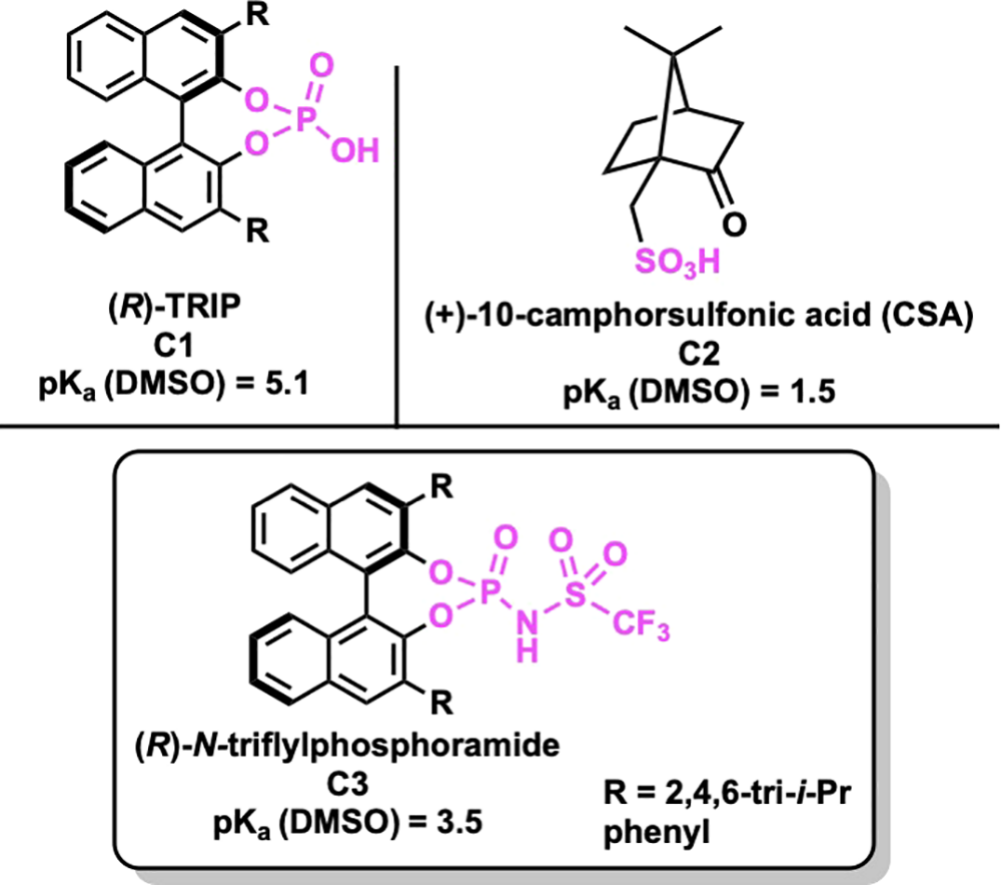
Catalysts investigated.

**Scheme 3:**
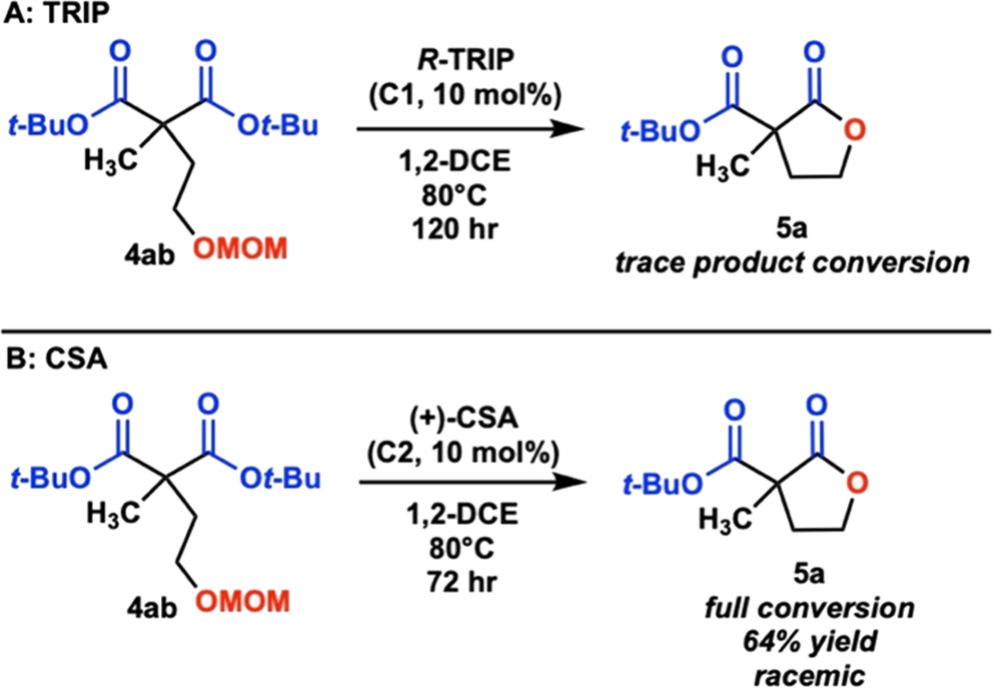
Initial Reactions with (A) TRIP (**C1**) and (B) CSA (**C2**)

Initially, we investigated the conversion of methyl-substituted MOM-protected alcohol **4ab** to lactone **5a** with 10 mol % of the chiral phosphoric acid TRIP, **C1**, which we had used in our previous work (Scheme 3A). After several days, though, only a trace conversion was seen. However, when reacted with 10 mol % of (+)-camphorsulfonic acid, **C2**, full conversion to **5a** was observed, but the product was racemic (Scheme 3B). Given these data, we hypothesized that a difference in p*K*_a_ between **C1** and **C2** led to the observed difference in reactivity and that a stronger chiral phosphoric acid catalyst might accomplish the transformation to **5a** (Figure 1), but still allow for enantioselectivity.

**Scheme 4:**
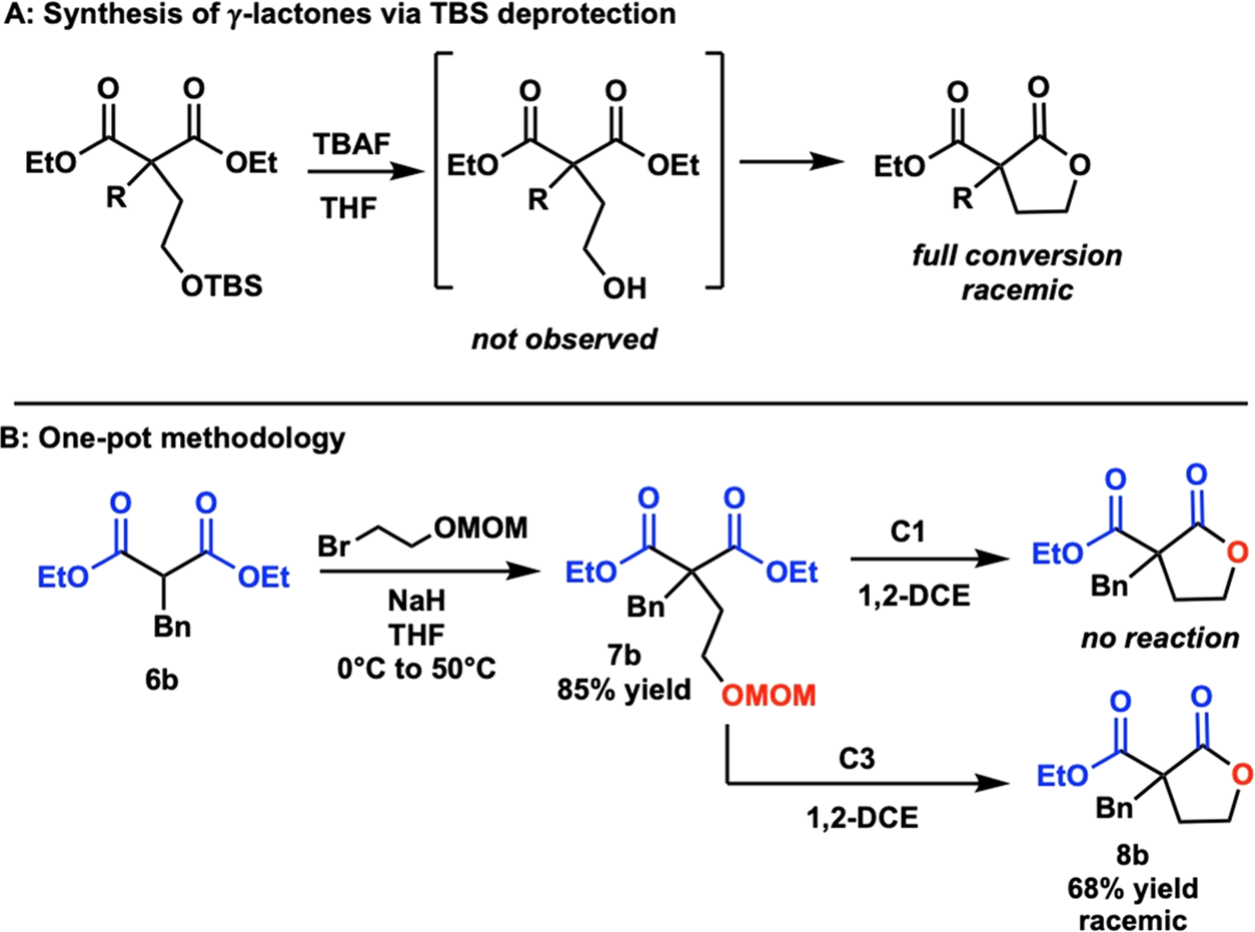
(A) Deprotection of Silyl Groups with TBAF. (B) Testing the One-Pot Methodology on Smaller Esters

We next evaluated smaller esters in a cascade to enantioenriched lactones. Previously, stereoselective cyclization of these esters was impossible with our desymmetrization, due to the rapid rate at which the deprotected alcohol reacts with smaller esters, even without the presence of an acid catalyst (Scheme 4A). For comparison, compound **7b** was readily synthesized from commercially available **6b**. With **C1**, no conversion was observed to **7b** and only led to recovery of the starting material. With **C3**, however, **7b** was able to convert to lactone **8b**, but no enantioenrichment of the product was observed (Scheme 4B). These data led us to the hypothesis that the rate-determining step of the cascade is the removal of the protecting group and that a stronger acid is required for protecting group removal.

**Figure 2:**
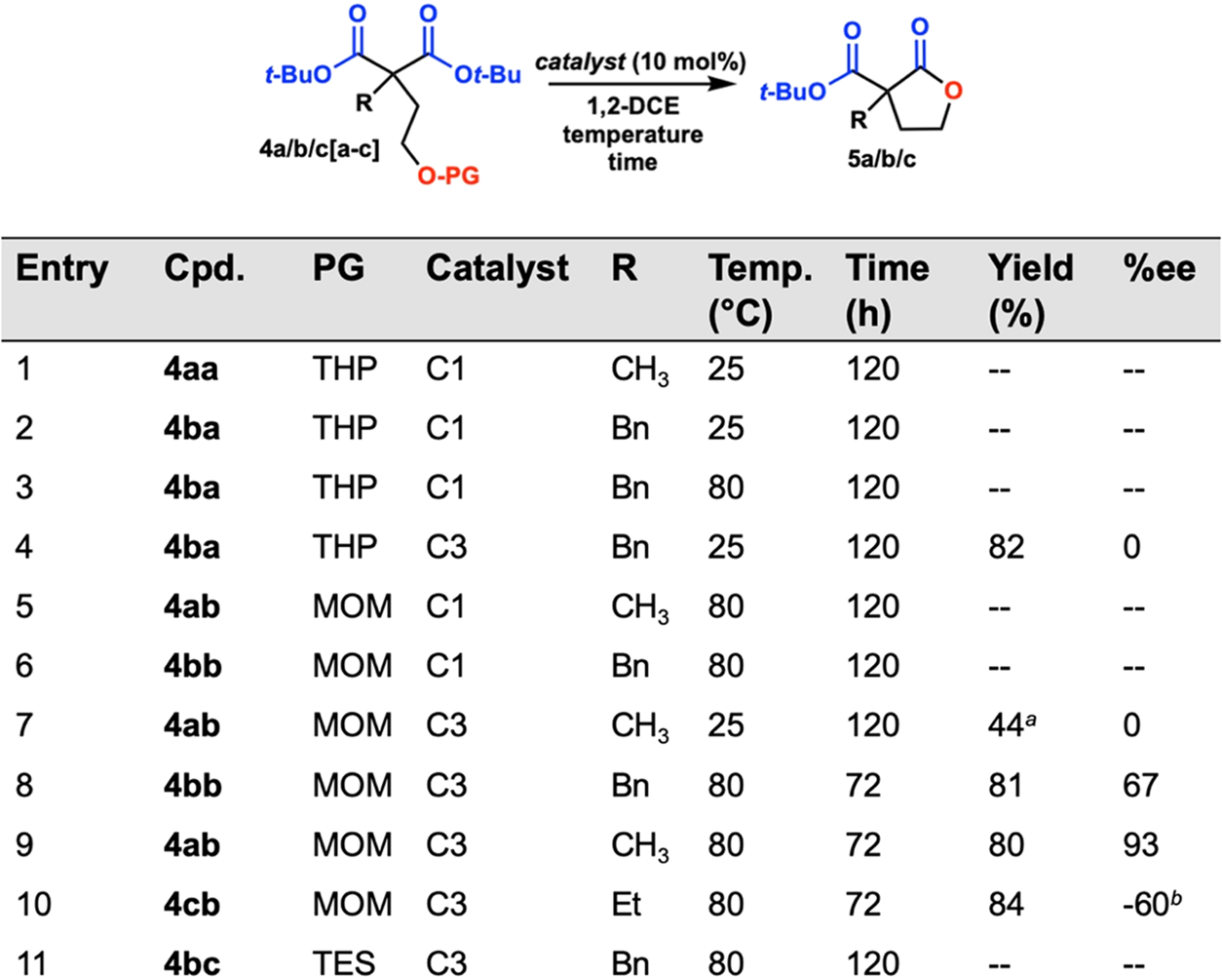
Protecting group reactions. (a) Gas chromatography (GC) yield and (b) opposite enantiomer catalyst used.

We next turned our attention to the *tert*-butyl esters required in our previous work and investigated the conversion of protected compounds **4a/b[a–c]** into lactones **5a/b** (Figure 2). Methyl and benzyl THP-protected alcohols **4aa** and **4ba** (Entries 1 and 2) did not lead to removal of the protecting group with **C1**, even at elevated temperatures (Entry 3), and only starting material was recovered. With the stronger acid **C3**, however, benzyl THP-protected alcohol **4ba** (Entry 4) saw full removal of the THP protecting group and cyclized to form lactone **5b**, but in racemic mixtures, even at room temperature (rt). Turning to the more stable MOM protecting group, with catalyst **C1** methyl or benzyl MOM-protected alcohols **4ab** and **4bb** were unable to form product **5a** or **5b** (Entries 5 and 6) with only starting material recovered, even at elevated temperatures. At room temperature, methyl MOM-protected alcohol **4ab** converted to **5a** with **C3** (Entry 7) only at a small scale (5 mg) and could not be isolated. Gratifyingly, however, methyl, ethyl, and benzyl MOM-protected alcohols **4ab** through **4cb** (Entries 8–10) were converted to their target lactones **5a** through **5c** with catalyst **C3** in good yields and good enantioselectivities when heated to 80 °C. Additionally, the benzyl TES-protected alcohol **4bc** was unable to be removed with **C3** (Entry 11) resulting in recovery of starting material.

**Scheme 5:**
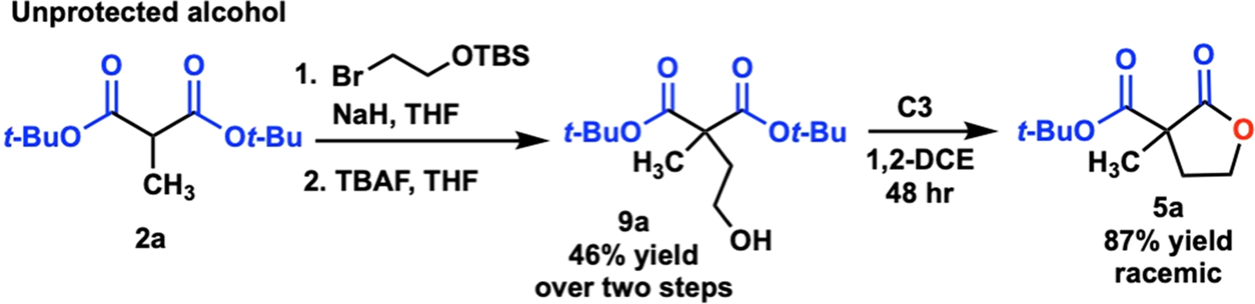
Testing New Catalyst on Unprotected Alcohol

Additionally, we tested catalyst **C3** on a previously published reaction from the Petersen group that achieved 98% *ee* with catalyst **C1**. To our surprise, catalyst **C3** readily converted **9a** to lactone **5a** utilizing our standard conditions (80 °C), but as a racemic mixture (Scheme 5). Furthermore, racemic mixtures were also obtained when reaction temperatures were lowered to both 25 and 0 °C.

**Figure 3:**
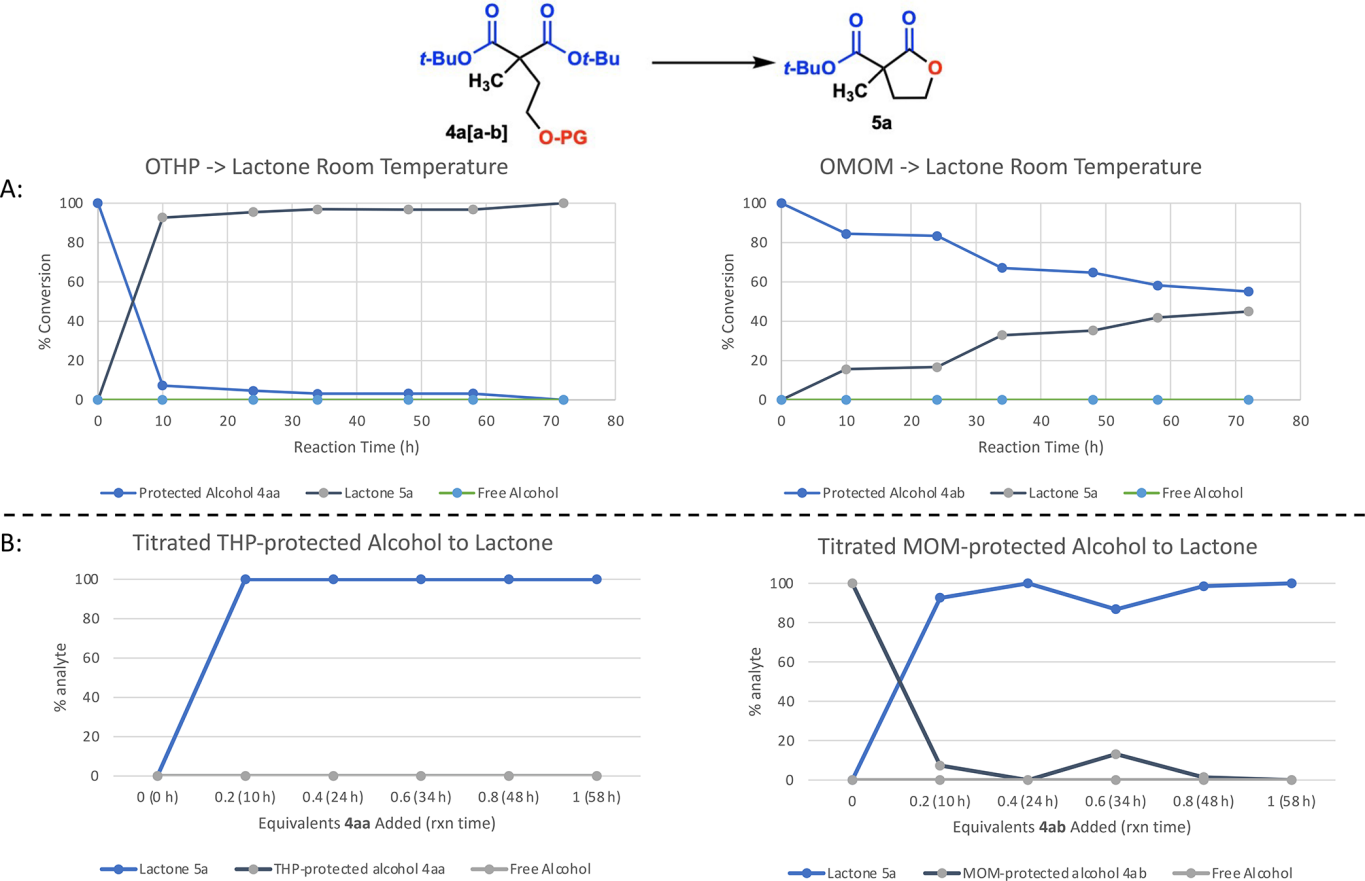
GC investigation of the reaction rate.

To further understand the mechanism and investigate the rate of deprotection, we continuously monitored the conversion of THP- and MOM-protected alcohols to their corresponding lactone **5a** via GC. At room temperature, THP-protected **4aa** converted readily to lactone **5a**, with 92% conversion at the 10 h mark. Meanwhile, MOM-protected **4ab** reacted significantly more slowly, reaching only 44% conversion to **5a** by 72 h (Figure 3A). Additionally, we performed an experiment where 0.2 equiv aliquots of **4aa/ab** were added sequentially over the course of 72 h to a solution of 10 mol % catalyst (relative to 1 equiv of **4aa/ab**) in 1,2-dichloroethene (DCE) at 80 °C. After each addition, reaction progress was measured via GC. With THP-protected **4aa**, we saw full conversion of each aliquot to lactone **5a** at each time point, pointing to a rapid rate of deprotection and cyclization. However, with MOM-protected **4ab**, conversion to **5a** of each aliquot was not complete, even at more stoichiometric catalyst–substrate ratios (Figure 3B). Finally, no concentration of free alcohol was detected while running either GC experiment, pointing to the cyclization step as the fastest step in the cascade. These data point to a slower rate of MOM deprotection in our reaction system, particularly when compared to that of THP-protected alcohols.

**Scheme 6:**
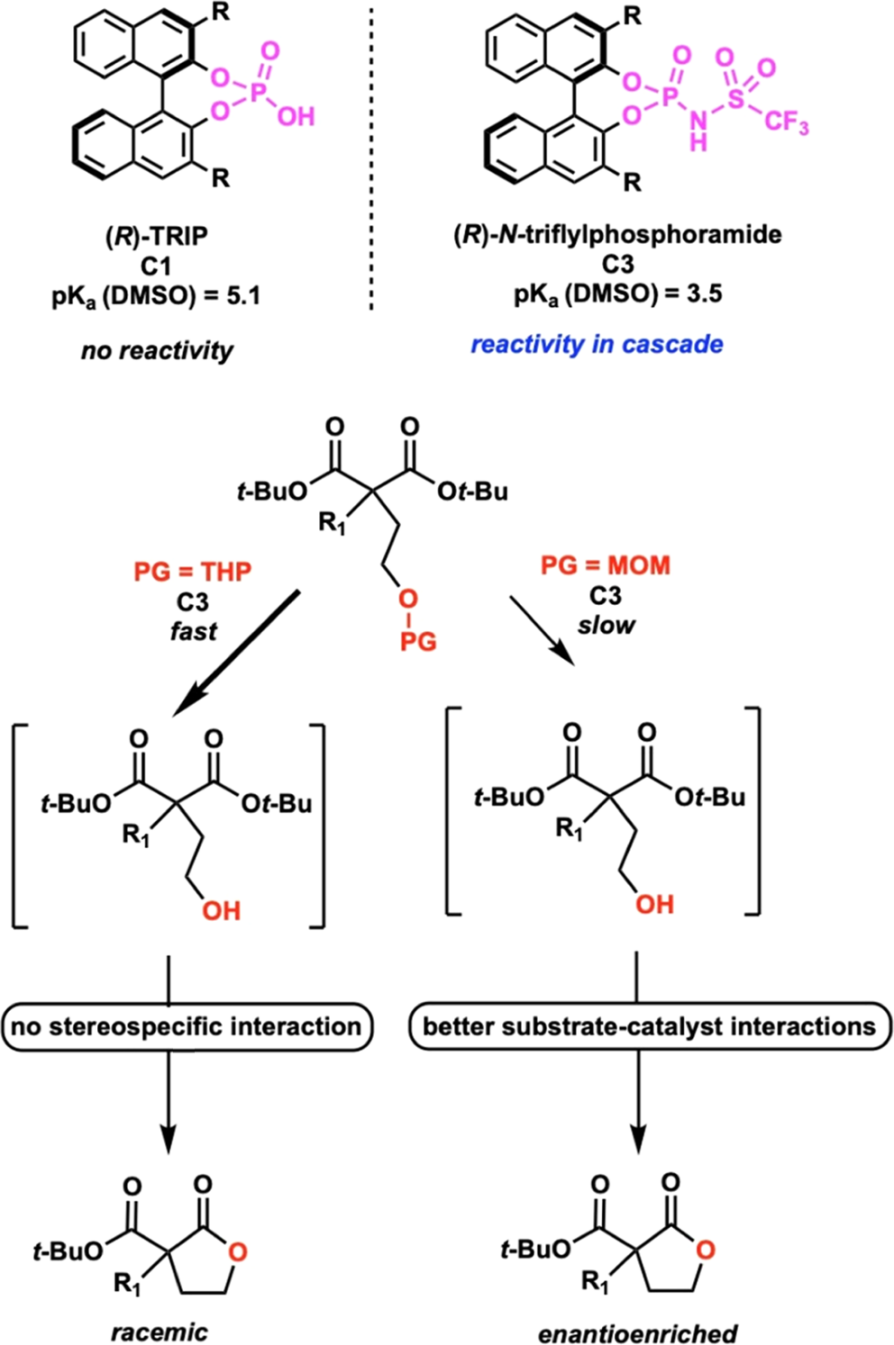
Hypothesized Rate Differences in Protecting Groups

Taken together, we hypothesize that the mechanism and the observed stereoselectivity rely on the acidity of the catalyst, the rate at which the catalyst both deprotects and cyclizes the substrate, and the protecting group used. We observed that THP protecting groups were more labile to acid cleavage than the MOM protecting groups, supported by our GC studies. This is further supported in the literature, where equivalent substrates with MOM- or THP-protected alcohols need longer or harsher conditions to deprotect MOM-protected alcohols versus THP-protected alcohols.^14,15^ We also hypothesize that the lower p*K*_a_ of **C3** with respect to **C1** causes a significant increase in the reaction rate, and with more labile THP-protected alcohols, this increased reaction rate does not allow enough time for the catalyst to selectively interact with the substrate (Scheme 6). Similarly, based on these data, we observe that the rate-determining step of this reaction is the initial deprotection step. Thus, with less labile MOM-protected substrates, the deprotection by the catalyst is slow enough to allow for a sufficient catalyst–substrate interaction, giving the observed stereoselectivity.

Here, we have presented a Brønsted acid catalyzed deprotection and stereoselective cyclization cascade reaction. To the best of our knowledge, this represents the first such catalytic cascade reaction where the chiral Brønsted acid catalyst acts to both remove the protecting group and stereoselectively introduce an intramolecular cyclization reaction. This Brønsted acid catalyzed deprotection cascade represents a promising new tool for asymmetric synthesis, and we have begun work to further elucidate the reaction mechanism and explore new applications for this cascade.

## Experimental Section

The data underlying this study are available in the published article and the Supporting Information. FAIR data are available as the Supporting Information for publication and includes the primary NMR FID files for compounds: **2a**, **2b**, **3a**, **3c**, **4aa**, **4ab**, **4ba**, **4bb**, **4bc**, **5a**, **5b**, **7b**, **8b**, **9a**, **4cb**, and **5c**.

### General Methods

Unless noted, all solvents and reagents were obtained from commercial sources and used without further purification; anhydrous solvents were dried following standard procedures. The ^1^H and ^13^C nuclear magnetic resonance (NMR) spectra were plotted on 400 and 500 MHz spectrometers using CDCl_3_ as a solvent at rt. The NMR chemical shifts (δ) are reported in parts per million. Abbreviations for ^1^H NMR: s = singlet, d = doublet, m = multiplet, b = broad, t = triplet, q = quartet, and p = pentet. The reactions were monitored by thin layer chromatography (TLC) using silica G F254 precoated plates. Flash chromatography was performed using flash grade silica gel (particle size: 40–63 μm, 230 × 400 mesh). Enantiomeric excess was determined by high-performance liquid chromatography (HPLC) analysis. High-resolution mass spectra were acquired on an Orbitrap XL MS system and Q Exactive Plus MS system. The specific rotations were acquired on an analytical polarimeter.

#### Compound **2a**

To a mixture of methyl malonic acid (0.6 g, 4.8 mmol) **1a** in diethyl ether (2.5 mL) was added 4-(dimethylamino)pyridine (0.06 g, 0.4 mmol), *t*-butyl alcohol (7.5 mL), and di-*t*-butyl dicarbonate (2.4 g, 10.7 mmol). The mixture was stirred at room temperature for 48 h, after which the reaction mixture was quenched with water (20 mL) and 1 M HCl (20 mL). The mixture was extracted with ethyl acetate (3 × 20 mL). The combined organic layers were washed with 0.5 M NaOH (2 × 20 mL). The organic layer was dried over magnesium sulfate and concentrated, affording the product **2a** as a colorless oil (687 mg, 61% yield). ^1^H NMR (500 MHz, CDCl_3_) δ 3.21 (q, *J* = 7.2 Hz, 1H), 1.45 (s, 18H), 1.31 (d, *J* = 7.3 Hz, 3H); ^13^C NMR (125 MHz, CDCl_3_) δ 169.7, 81.3, 48.2, 27.9, 13.5. Data match previously reported.^13^

#### Compound **3a**

To a solution of 2-bromoethanol (1.5 mL, 21.2 mmol) in CH_2_Cl_2_ (21 mL) at 0 °C was added pyridinium *p*-toluene sulfonate (0.5 g, 2.1 mmol). 3,4-Dihydro-2*H*-pyran (2.9 mL, 31.8 mmol) was added, and the reaction mixture was allowed to warm to room temperature for 16 h. The reaction was quenched with 20 mL of deionized water and extracted with CH_2_Cl_2_ (3 × 25 mL). The combined organic layers were dried over magnesium sulfate and concentrated. The crude oil was purified via flash chromatography (10% ethyl acetate in hexanes) to afford **3a** as a colorless oil (4.23 g, 92% yield). ^1^H NMR (400 MHz, CDCl_3_) δ 4.65 (m, 1H), 3.99 (m, 1H), 3.86 (m, 1H), 3.74 (m, 1H), 3.49 (m, 3H), 1.47–1.88 (br m, 6H); ^13^C NMR (100 MHz, CDCl_3_) δ 99.0, 67.6, 62.3, 30.9, 29.9, 25.4, 19.3. Data match previously reported.^16^

#### Compound **3c**

To a solution of 2-bromoethanol (0.3 mL, 4.4 mmol) in CH_2_Cl_2_ (10 mL) at room temperature was added triethylamine (1.5 mL, 11.1 mmol) and chlorotriethylsilane (0.7 mL, 4.43 mmol). The reaction mixture was stirred for 16 h. The reaction was quenched with 10 mL of deionized water and extracted with ethyl acetate (3 × 20 mL), and the combined organic layers were dried over magnesium sulfate and concentrated to afford **3c** as a colorless oil (1.01 g, 95% yield). ^1^H NMR (400 MHz, CDCl_3_) δ 3.87 (t, *J* = 6.7 Hz, 2H), 3.39 (t, *J* = 6.6 Hz, 2H), 0.95 (t, *J* = 7.9 Hz, 9H), 0.61 (q, *J* = 8.0 Hz, 6H); ^13^C NMR (100 MHz, CDCl_3_) δ 63.3, 33.2, 6.7, 4.4. Data match previously reported.^17^

### Typical Procedure for Alkylation Reactions

To a solution of malonate starting material (1.1 equiv) in THF (0.3 M) in an ice bath is added NaH (60% dispersion in mineral oil, 2 equiv). After 5 min, the alkylating agent (1 equiv) is added, and the reaction mixture moved to a 50 °C oil bath and allowed to stir for 24 h, or until completion is observed via TLC. The reaction mixture is quenched with a saturated brine solution (20 mL) and extracted with ethyl acetate (3 × 20 mL), and the combined organic layers were dried over magnesium sulfate and concentrated. The crude material was purified via flash column chromatography (10% ethyl acetate in hexanes) to afford alkylated malonate.

#### Compound **2b**

Compound **2b** is a colorless oil (1.23 g, 69% yield); ^1^H NMR (400 MHz, CDCl_3_) δ 7.28 (m, 3H), 7.19 (m, 2H), 3.45 (t, *J* = 8 Hz, 1H), 3.11 (d, *J* = 8 Hz, 2H), 1.39 (s, 18H); ^13^C NMR (100 MHz, CDCl_3_) δ 168.4, 138.3, 129.0, 128.4, 126.5, 81.6, 55.6, 34.6, 27.9. Data match previously reported.^12^

#### Compound **4aa**

Compound **4aa** is a yellowish oil (125 mg, 73% yield); ^1^H NMR (400 MHz, CDCl_3_) δ 4.56 (m, 1H), 3.80 (m, 2H), 3.45 (m, 2H), 2.13 (m, 2H), 1.74 (m, 2H), 1.55 (m, 2H), 1.49 (m, 2H), 1.47 (s, 3H), 1.44 (s, 18H); ^13^C NMR (100 MHz, CDCl_3_) δ 171.5, 98.9, 81.1, 63.7, 62.2, 53.3, 34.9, 30.6, 27.9, 25.5, 19.9, 19.5; HRMS (C_19_H_34_O_6_, ESI) calcd 359.2428 [M + H]^+^, found 359.2424.

#### Compound **4ab**

Compound **4ab** is a colorless oil (105 mg, 85% yield); ^1^H NMR (400 MHz, CDCl_3_) δ 4.54 (s, 2H), 3.55 (t, *J* = 7.0 Hz, 2H), 3.33 (s, 3H), 2.11 (t, *J* = 7.0 Hz, 2H), 1.43 (s, 18H), 1.33 (s, 3H); ^13^C NMR (100 MHz, CDCl_3_) δ 171.4, 96.3, 81.1, 63.7, 55.3, 53.2, 34.9, 27.9, 19.7; HRMS (C_16_H_30_O_6_, ESI) calcd 341.1935 [M + Na]^+^, found 341.1934.

#### Compound **4ba**

Compound **4ba** is a colorless oil (171 mg, 81% yield); ^1^H NMR (400 MHz, CDCl_3_) δ 7.24 (m, 3H), 7.18 (m, 2H), 4.66 (m, 1H), 4.00 (m, 1H), 3.88 (m, 1H), 3.75 (m, 1H), 3.50 (m, 3H), 3.11 (d, *J* = 7.1 Hz, 2H), 1.83 (m, 1H), 1.72 (m, 2H), 1.53 (m, 4H), 1.39 (s, 18H); ^13^C NMR (100 MHz, CDCl_3_) δ 168.3, 138.3, 129.0, 128.4, 126.5, 99.0, 81.6, 67.6, 62.3, 55.6, 34.6, 27.9, 27.7, 25.4, 19.3; HRMS (C_25_H_38_O_6_, ESI) calcd 435.2741 [M + H]^+^, found 435.2743.

#### Compound **4bb**

Compound **4bb** is a colorless oil (543 mg, 93% yield); ^1^H NMR (400 MHz, CDCl_3_) δ 7.20 (m, 5H), 4.57 (s, 2H), 3.61 (t, *J* = 7.0 Hz, 2H), 3.34 (s, 3H), 3.21 (s, 2H), 2.04 (t, *J* = 6.9 Hz, 2H), 1.44 (s, 18H); ^13^C NMR (100 MHz, CDCl_3_) δ 170.3, 136.5, 130.3, 128.2, 126.8, 96.3, 81.6, 63.7, 57.9, 55.3, 38.0, 31.5, 27.9; HRMS (C_22_H_34_O_6_, ESI) calcd 395.2428 [M + H]^+^, found 395.2430.

#### Compound **4bc**

Compound **4bc** is a colorless oil (159 mg, 82% yield); ^1^H NMR (400 MHz, CDCl_3_) δ 7.22 (m, 5H), 3.66 (t, *J* = 7.6 Hz, 2H), 3.19 (s, 2H), 2.00 (t, *J* = 7.6 Hz, 2H), 1.43 (s, 18H), 0.93 (t, *J* = 7.9 Hz, 9H), 0.57 (q, *J* = 8.0 Hz, 6H); ^13^C NMR (100 MHz, CDCl_3_) δ 170.3, 136.7, 130.4, 128.1, 126.7, 81.5, 59.3, 58.0, 38.4, 34.4, 27.9, 6.8, 4.3; HRMS (C_26_H_44_O_5_Si, ESI) calcd 465.3031 [M + H]^+^, found 465.3033.

#### Compound **4cb**

Compound **4cb** is a yellowish oil (268 mg, 63% yield); ^1^H NMR (400 MHz, CDCl_3_) δ 4.53 (s, 2H), 3.49 (t, 2H), 3.32 (s, 3H), 2.12 (t, 2H), 1.86 (q, 2H), 1.42 (s, 1H), 0.80 (t, 3H); ^13^C NMR (100 MHz, CDCl_3_) δ 170.9, 96.3, 81.1, 63.5, 57.2, 55.3, 30.9, 27.9, 24.9, 8.4; HRMS (C_17_H_32_O_6_, ESI) calcd 332.2199 [M + H]^+^, found 332.2197.

#### Compound **7b**

Compound **7b** is a yellowish oil (848 mg, 85% yield); ^1^H NMR (400 MHz, CDCl_3_) δ 7.22 (m, 3H), 7.09 (m, 2H), 4.55 (s, 2H), 4.16 (q, *J* = 7.2 Hz, 4H), 3.62 (t, *J* = 6.7 Hz, 2H), 3.34 (s, 3H), 3.28 (s, 2H), 2.10 (t, *J* = 6.6 Hz, 2H), 1.23 (t, *J* = 7.1 Hz, 6H); ^13^C NMR (100 MHz, CDCl_3_) δ 171.1, 136.1, 130.1, 128.3, 127.0, 96.4, 63.6, 61.3, 57.1, 55.3, 38.5, 31.8, 14.0; HRMS (C_18_H_26_O_6_, ESI) calcd 339.1802 [M + H]^+^, found 339.1806.

#### Compound **9a**

Compound **9a** is a colorless oil (651 mg, 46% yield); ^1^H NMR (400 MHz, CDCl_3_) δ 3.70 (t, *J* = 6.4 Hz, 2H), 2.04 (t, *J* = 6.4 Hz, 2H), 1.44 (s, 18H), 1.36 (s, 3H); ^13^C NMR (100 MHz, CDCl_3_) δ 172.1, 81.6, 59.0, 53.7, 38.2, 27.9, 20.2. Data match previously reported.^12^

### Typical Procedure for Deprotection/Cyclization Reactions

To a solution of dialkylated protected starting material (1 equiv) in 1,2-dichloroethane (0.025 M) at room temperature is added a catalyst (**C1**–**C3**, 0.1 equiv) and transferred to an 80 °C oil bath and stirred for 72 h or until reaction completion is determined by TLC analysis. The reaction mixture is quenched with deionized water (10 mL) and extracted with CH_2_Cl_2_ (3 × 10 mL). The combined organic layers are washed with brine (1 × 10 mL), dried over magnesium sulfate, and concentrated. The crude material was purified via flash column chromatography (20% ethyl acetate in hexanes) to afford the cyclized product.

#### Compound **5a**

Compound **5a** is a colorless oil (10 mg, 80% yield); ^1^H NMR (400 MHz, CDCl_3_) δ 4.32 (m, 2H), 2.66 (m, 1H), 2.15 (m, 1H), 1.45 (s, 12H); ^13^C NMR (100 MHz, CDCl_3_) δ 176.4, 169.5, 82.9, 65.9, 50.5, 35.2, 27.8, 20.1; 93% ee; [α]_D_^23^ = −2.9 (*c* = 1.1, CHCl_3_). Data match previously reported.^12^

#### Compound **5b**

Compound **5b** is a yellowish oil (11 mg, 81% yield); ^1^H NMR (400 MHz, CDCl_3_) δ 7.26 (m, 3H), 7.18 (m, 2H), 4.20 (q, *J* = 8.2 Hz, 1H), 3.84 (td, *J* = 8.7, 4.1 Hz, 1H), 3.30 (m, 2H), 2.62 (m, 1H), 2.27 (m, 1H); ^13^C NMR (100 MHz, CDCl_3_) δ 174.8, 170.0, 135.3, 130.1, 128.8, 127.5, 66.3, 55.5, 53.4, 39.3, 30.5; 67% ee; [α]_D_^23^ = +15.1 (*c* = 1.7, CHCl_3_). Data match previously reported.^12^

#### Compound **5c**

Compound **5c** is a yellowish oil (x mg, *x* yield); ^1^H NMR (400 MHz, CDCl_3_) δ 4.30 (m, 2H), 2.64 (m, 1H), 2.18 (m, 1H), 2.05 (m, 1H), 1.79 (m, 1H), 1.46 (s, 9H), 0.95 (t, 3H); 60% ee; [α]_D_^23^ = −1.1 (*c* = 1.9, CHCl_3_). Data match previously reported.^12^ Opposite enantiomer catalyst used.

#### Compound **8b**

Compound **8b** is a colorless oil (14 mg, 68% yield); ^1^H NMR (400 MHz, CDCl_3_) δ 7.28 (m, 3H), 7.20 (m, 2H), 4.26 (m, 2H), 3.85 (td, *J* = 8.7, 4.0 Hz, 1H), 3.29 (m, 2H), 2.62 (m, 1H), 2.28 (m, 1H), 1.30 (t, *J* = 7.1 Hz, 3H); ^13^C NMR (100 MHz, CDCl_3_) δ 175.0, 169.6, 135.4, 130.1, 128.8, 127.5, 66.3, 62.5, 55.5, 39.1, 30.6, 14.1; HRMS (C_14_H_16_O_4_, ESI) calcd 249.1121 [M + H]^+^, found 249.1116.

### Gas Chromatography Experiments

GC conditions: column: Agilent 19091G-B213; 0 m × 320 μm × 0.25 μm; flow rate: 1 mL/min; temperature ramp: 75 °C for 5 min, ramp 15 °C/min → 300 °C, 300 °C for 30 min.

#### Rate Experiment

For the rate experiment, a 15 mg sample of **4aa** or **4ab** was dissolved in DCE and 10 mol % **C3** was added, and the reaction was allowed to proceed at room temperature. A GC sample was taken at 0, 10, 24, 34, 48, 58, and 72 h to assess overall conversion. Controls of **4aa/ab**, **5a**, and **9a** were used as the standards in the experiment.

#### Titration Experiment

For the titration experiment, a sample of 10 mol % (relative to 15 mg of **4aa/ab**) was dissolved in DCE and allowed to stir at 80 °C. 0.2 equiv of **4aa** or **4ab** was added every 12 h, up to 1 equiv of **4aa/ab**. GC samples were taken before addition of **4aa/ab** and before every subsequent addition of 0.2 equiv.

## Supporting Information Available

^1^H NMR and ^13^C NMR spectra of synthesized compounds (Figures S1–S31) and racemic HPLC trace and enantioenriched HPLC trace for compounds **5a**, **5b**, and **5c** (Figures S32–S37) (PDF)
