# Supplementary material for: Chiral Brønsted Acid Catalyzed Cascade Alcohol Deprotection and Enantioselective Cyclization
Source: ACS Omega. 2023 Dec 29;9(2):2962–9. doi: 10.1021/acsomega.3c08869 (PMC10795110; doi:10.1021/acsomega.3c08869)
Supplement: Supplementary file 1 — ao3c08869_si_001.pdf [file ao3c08869_si_001.pdf]

# Chiral Brønsted Acid Catalyzed Cascade Alcohol Deprotection and Enantioselective Cyclization

Joshua A. Frost, Sarah M. Korb, Fiona E. Green, Kala C. Youngblood, Kimberly S. Petersen.

Department of Chemistry and Biochemistry
University of North Carolina at Greensboro
Greensboro, NC 27405

Table of Contents, Supporting Information:

Copies of NMR Spectra…………………………………………………………… S2-S17
Copies of Chiral HPLC Chromatograms…………………………………………… S18-S20

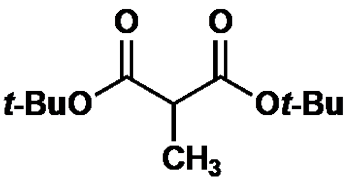


**2a**

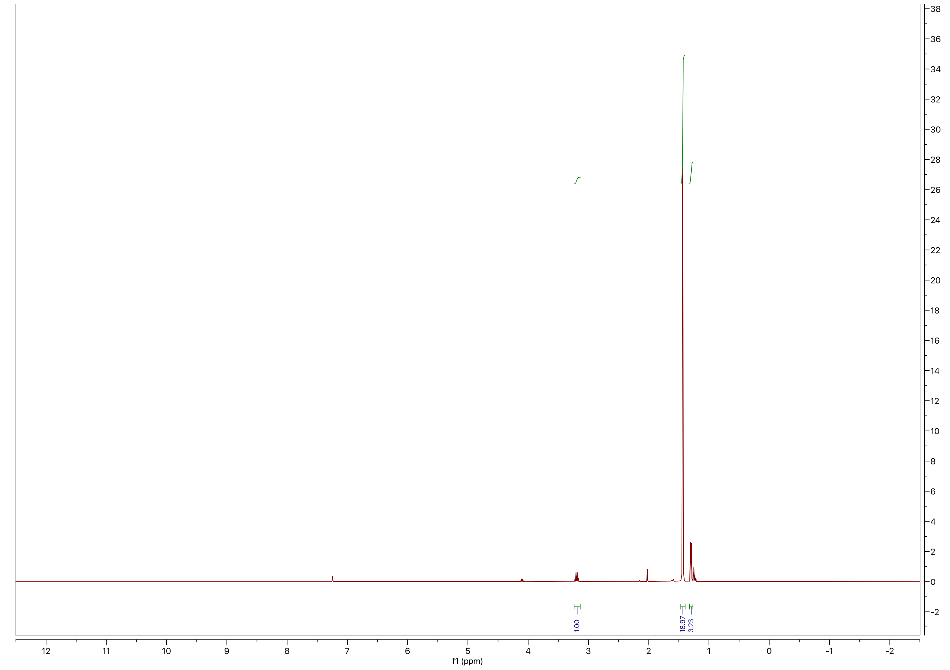


**Figure S1.** $^{1}H$ NMR spectrum of **2a**.

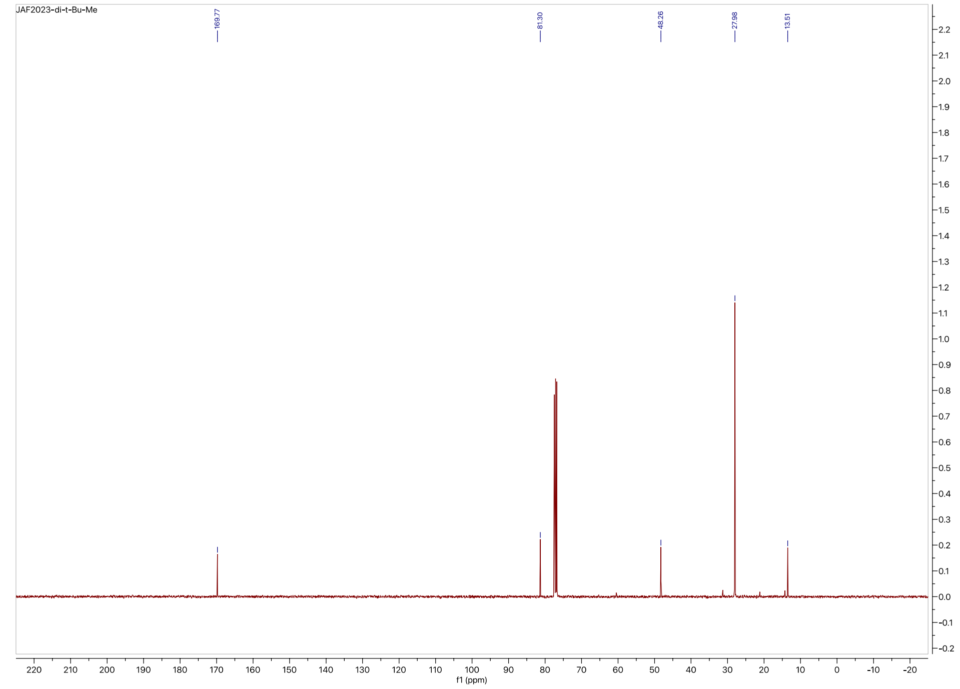


**Figure S2.** $^{13}C$ NMR spectrum of **2a**.

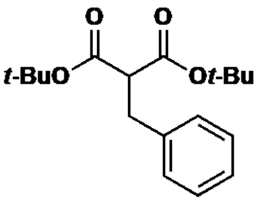


**2b**

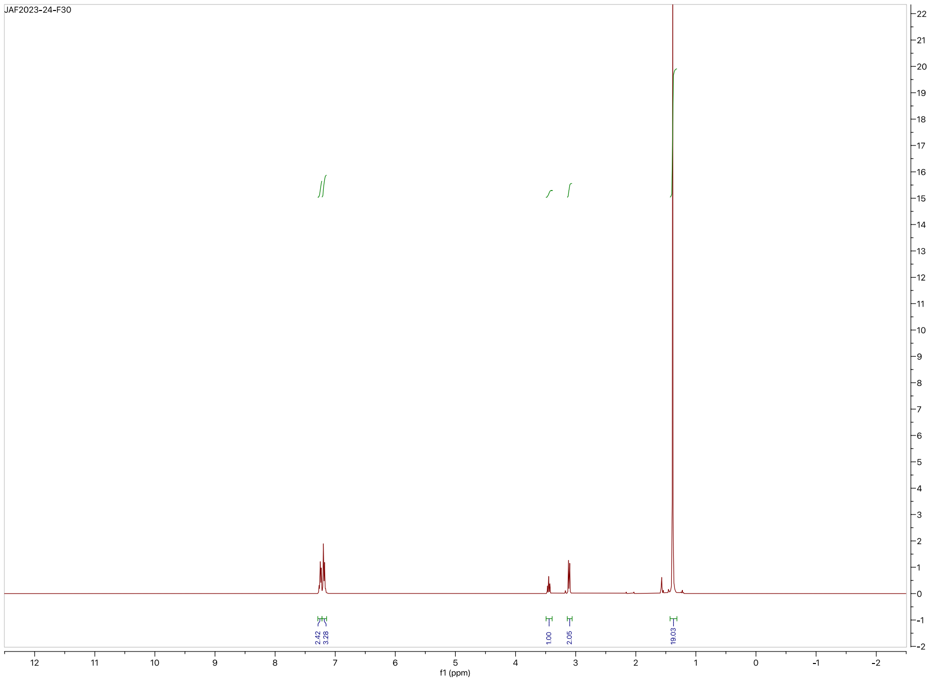


**Figure S3.** $^{1}H$ NMR spectrum of **2b**.

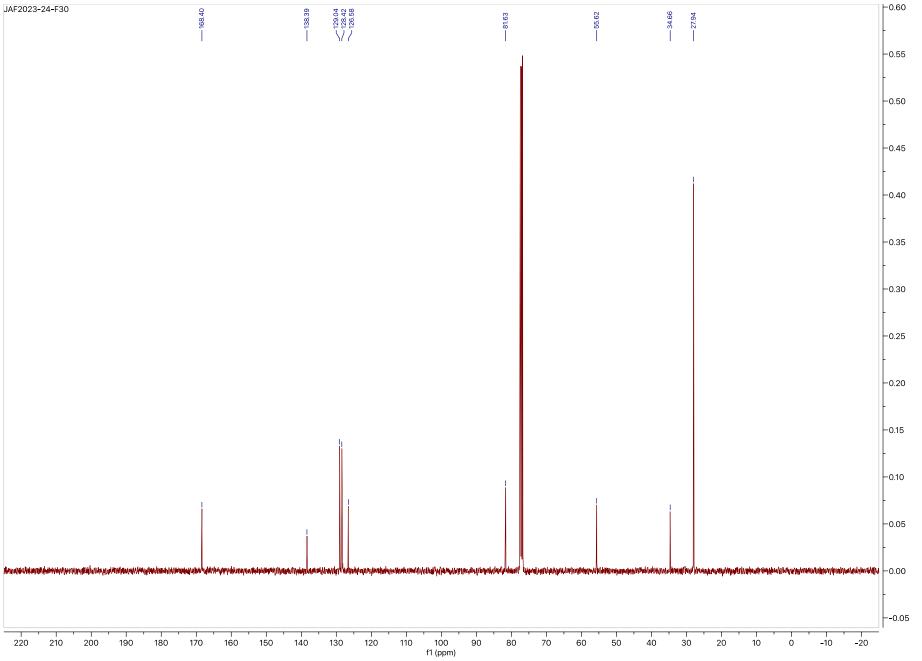


**Figure S4.** $^{13}C$ NMR spectrum of **2a**.

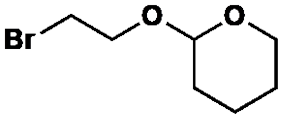


**3a**

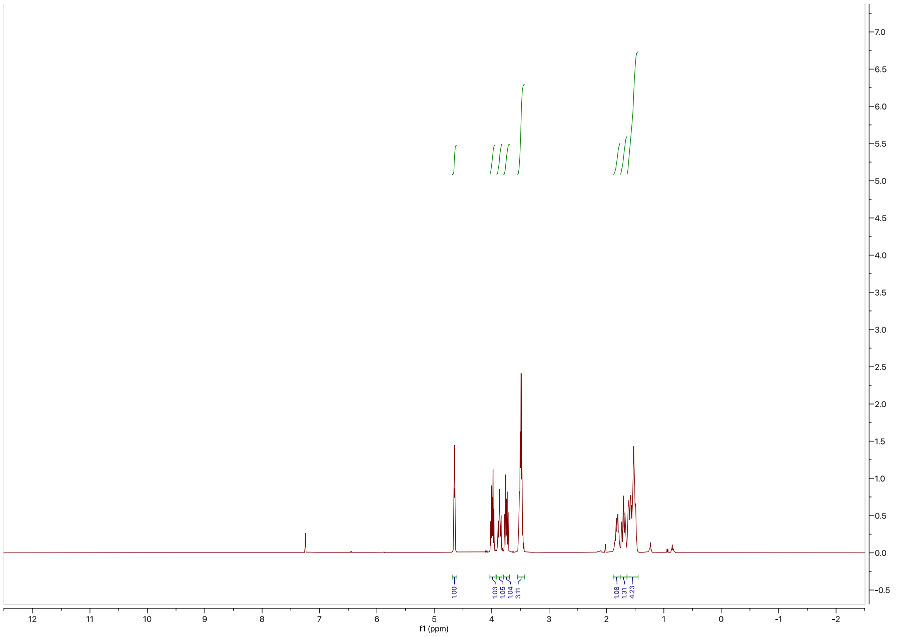


**Figure S5.** $^{1}H$ NMR spectrum of **3a**.

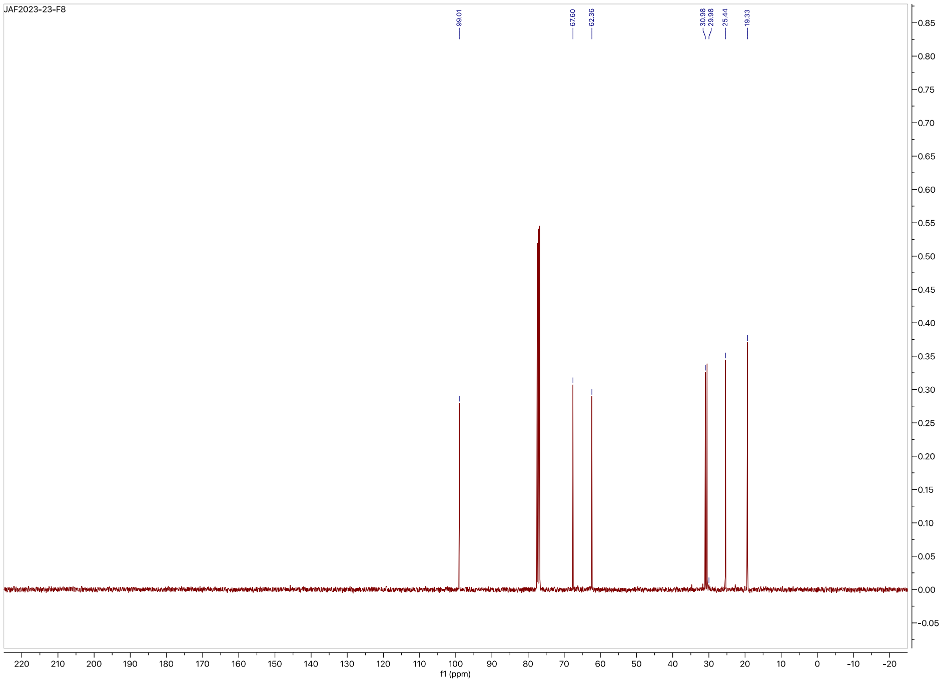


**Figure S6.** $^{13}C$ NMR spectrum of **3a**.

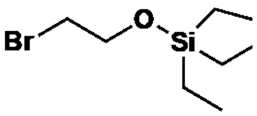


3c

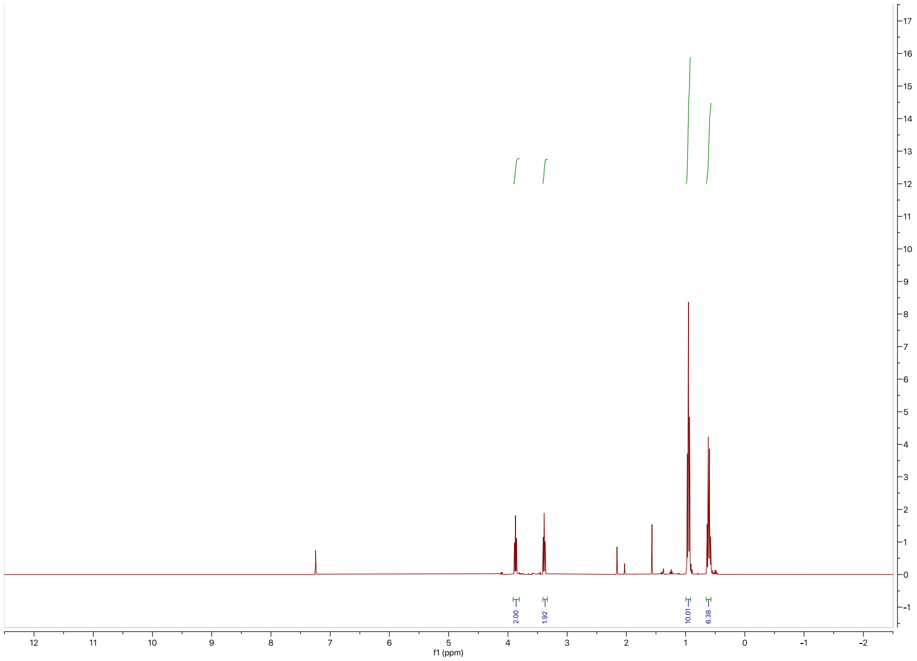

**Figure S7.** $^{1}H$ NMR spectrum of **3c**.

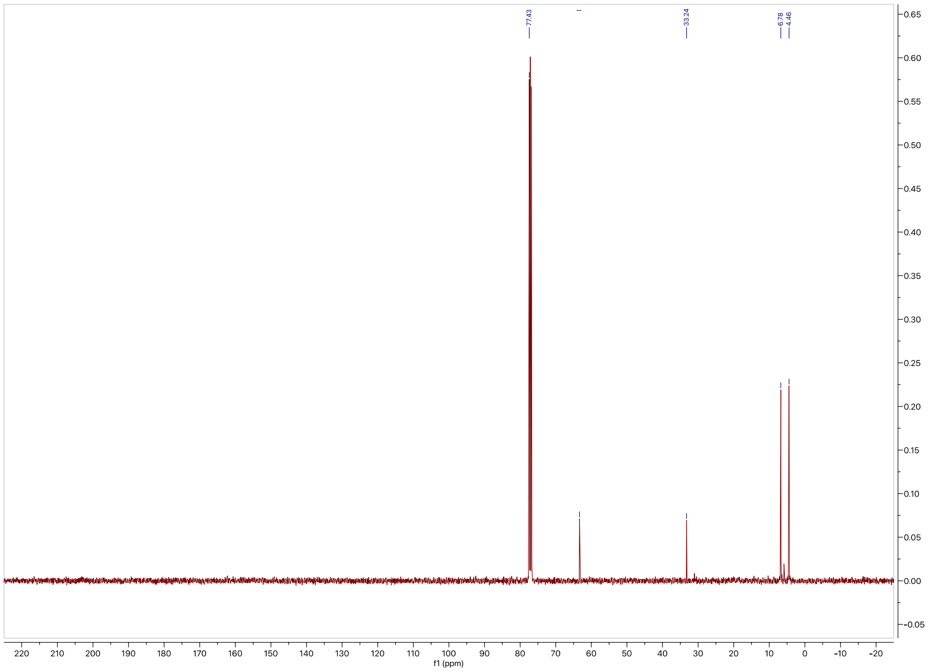

**Figure S8.** $^{13}C$ NMR spectrum of **3c**.

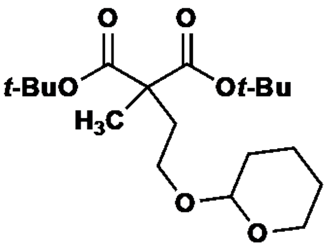

4aa

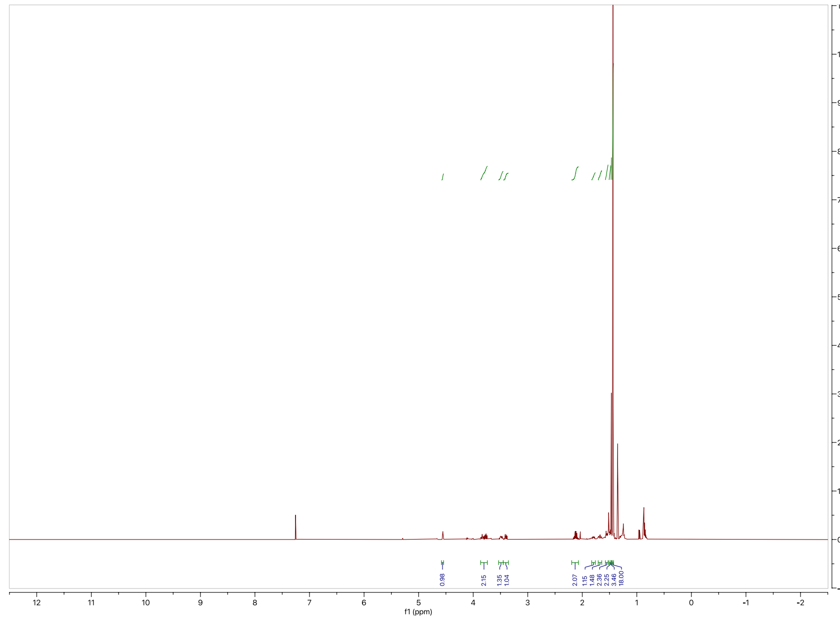

**Figure S9.** $^{1}$H NMR spectrum of **4aa**.

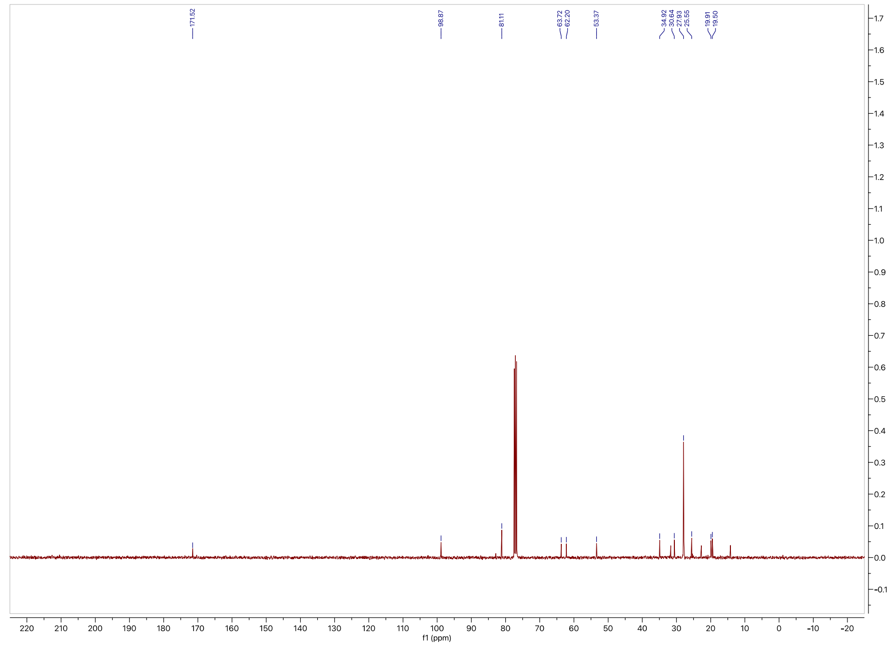

**Figure S10.** $^{13}$C NMR spectrum of **4aa**.

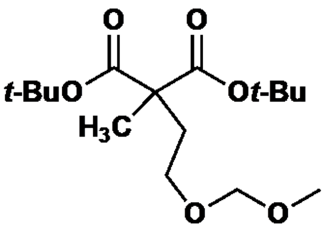


**4ab**

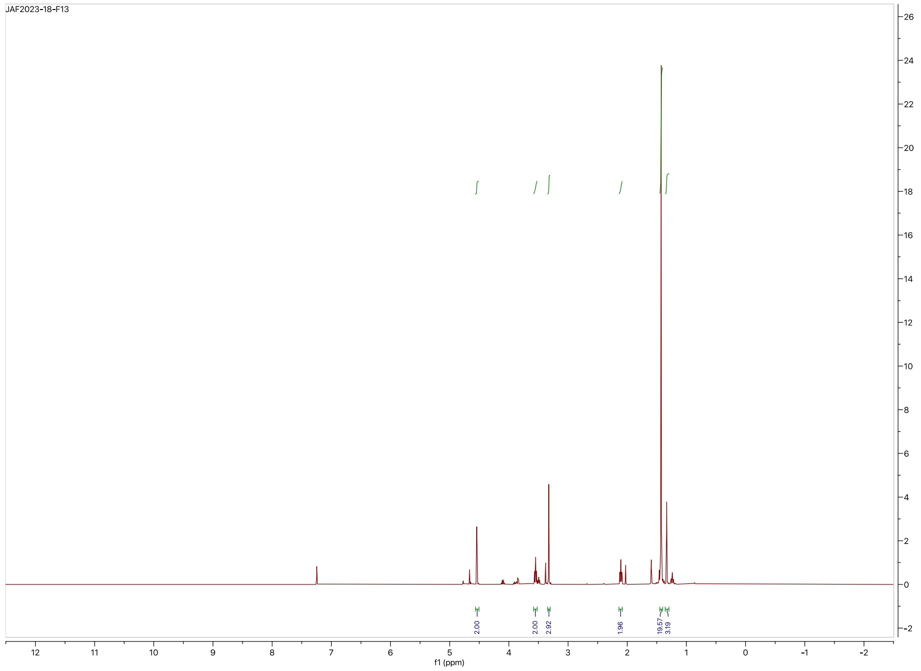


**Figure S11.** $^{1}$H NMR spectrum of **4ab**.

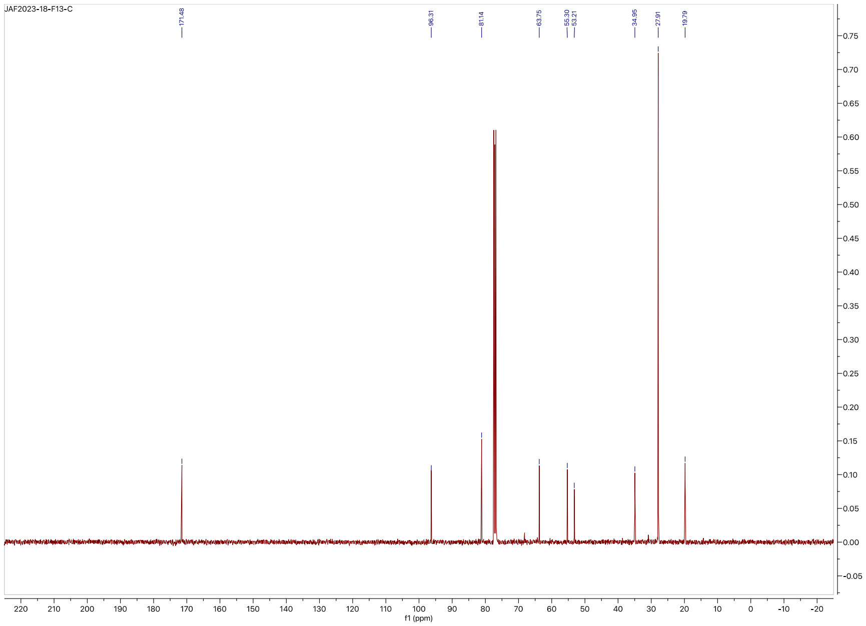


**Figure S12.** $^{13}$C NMR spectrum of **4ab**.

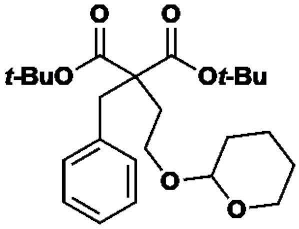


**4ba**

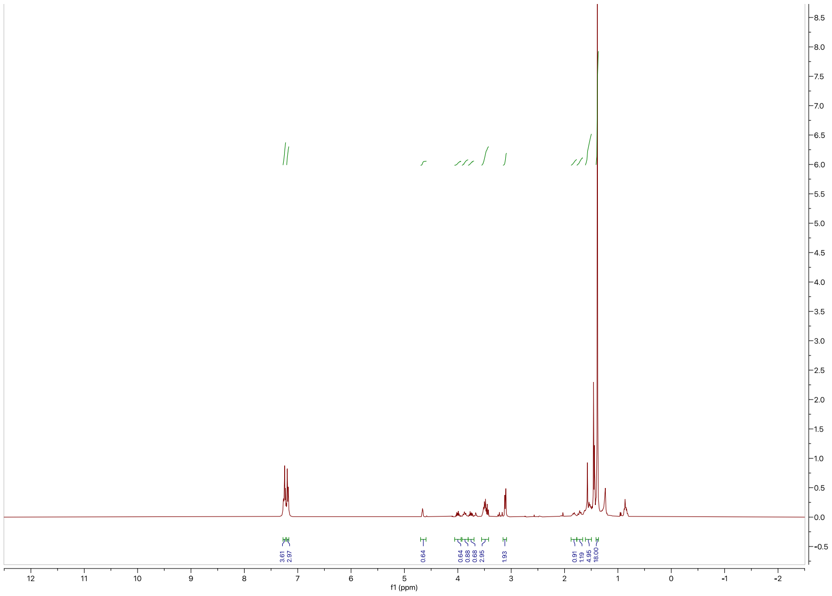

**Figure S13.** $^{1}$H NMR spectrum of **4ba**.

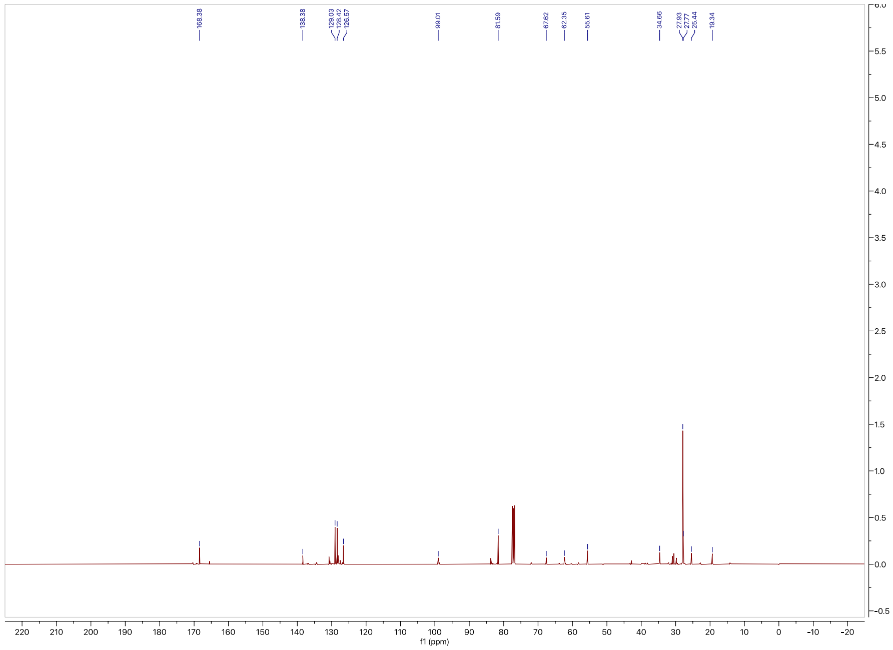

**Figure S14.** $^{13}$C NMR spectrum of **4ba**.

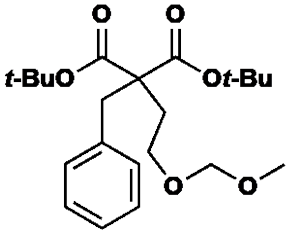


**4bb**

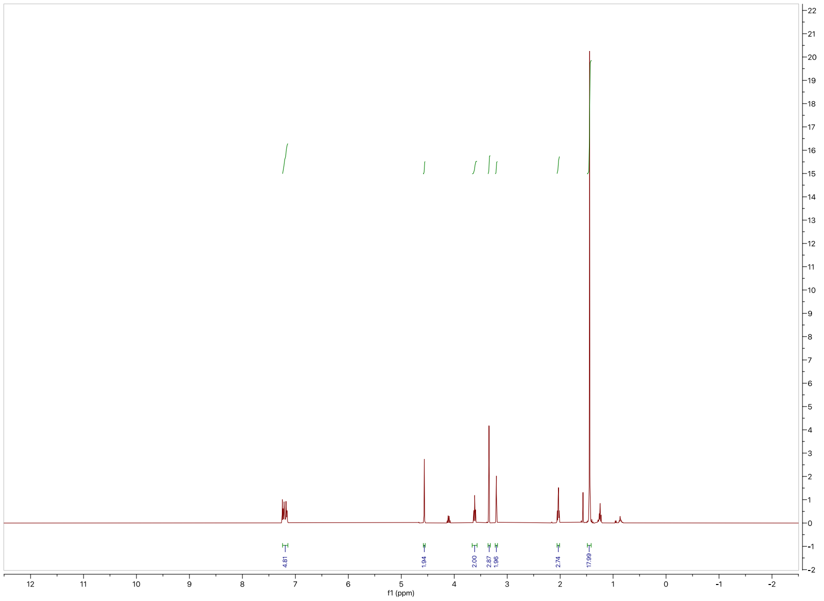


**Figure S15.** $^{1}$H NMR spectrum of **4bb**.

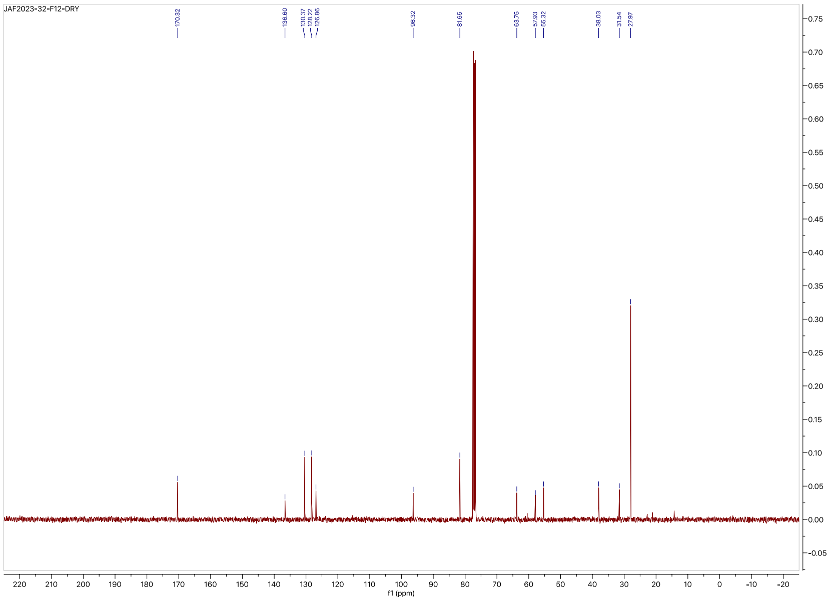


**Figure S16.** $^{13}$C NMR spectrum of **4bb**.

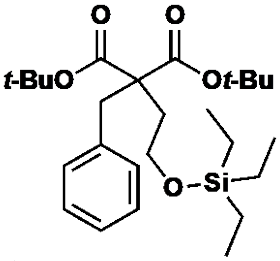

**4bc**

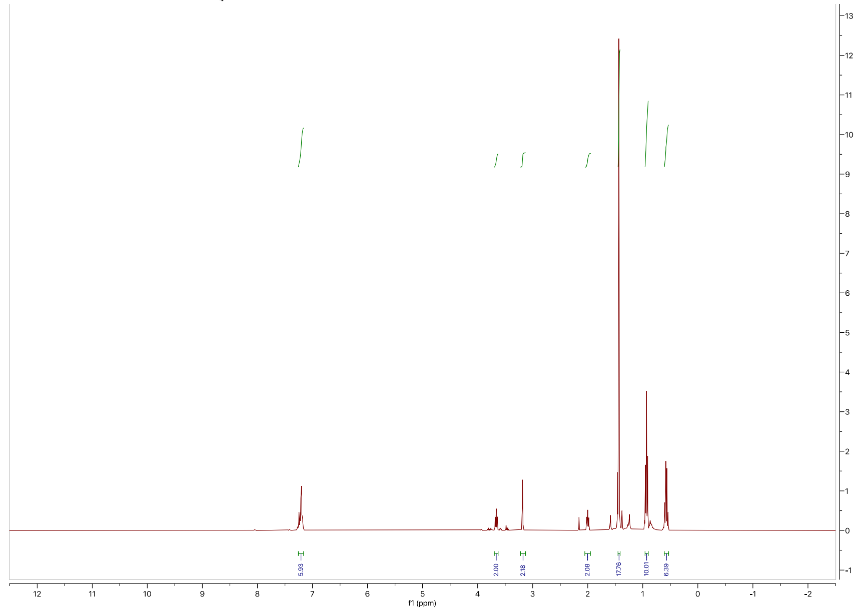


**Figure S17.** $^{1}$H NMR spectrum of **4bc**.

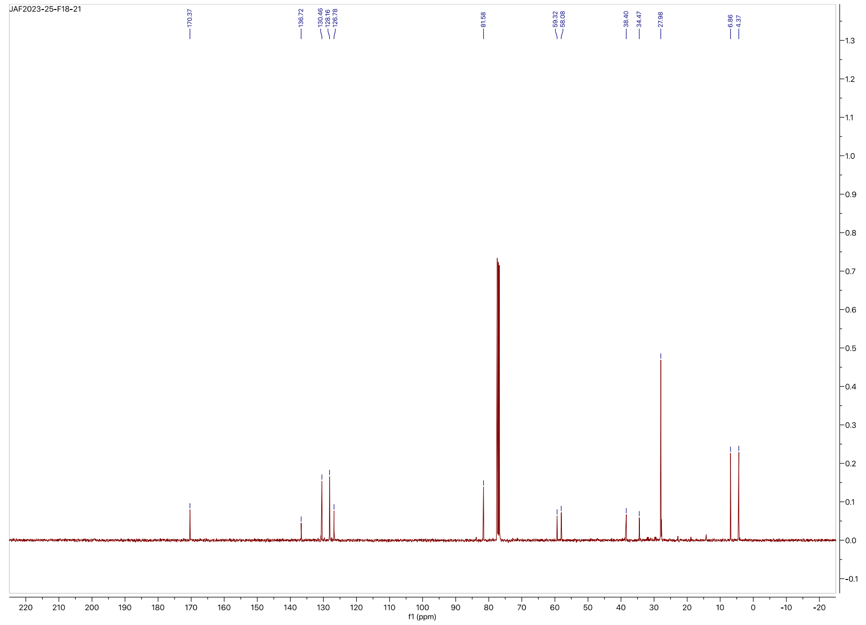


**Figure S18.** $^{13}$C NMR spectrum of **4bc**.

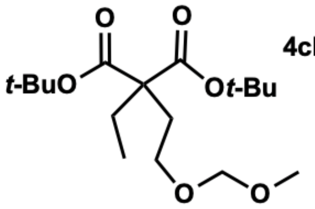

4cb

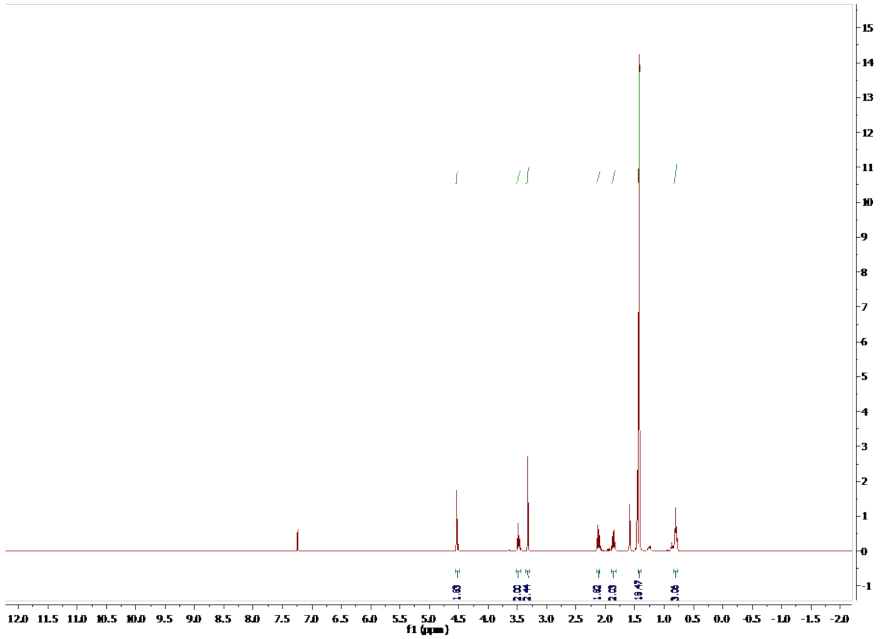

**Figure S19.** $^{1}H$ NMR spectrum of **4cb**.

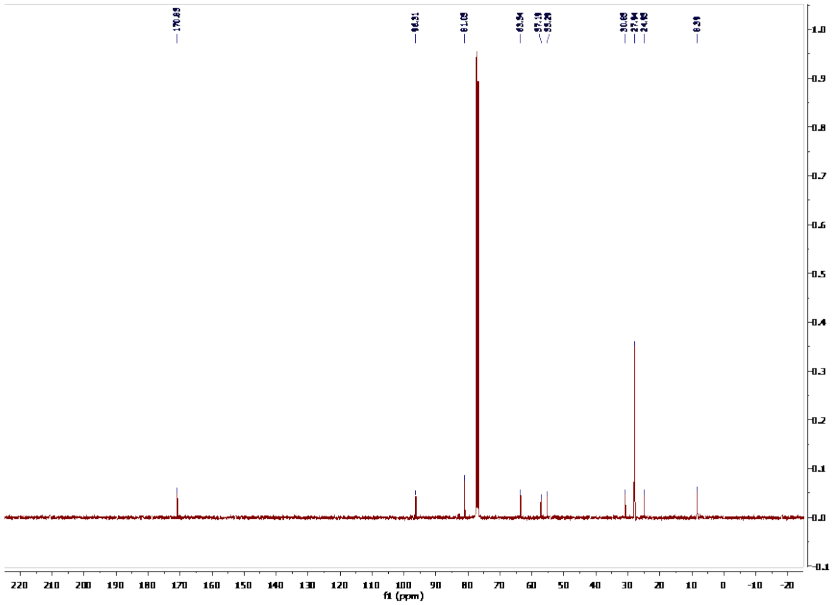

**Figure S20.** $^{13}C$ NMR spectrum of **4cb**.

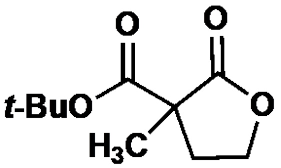


**5a**

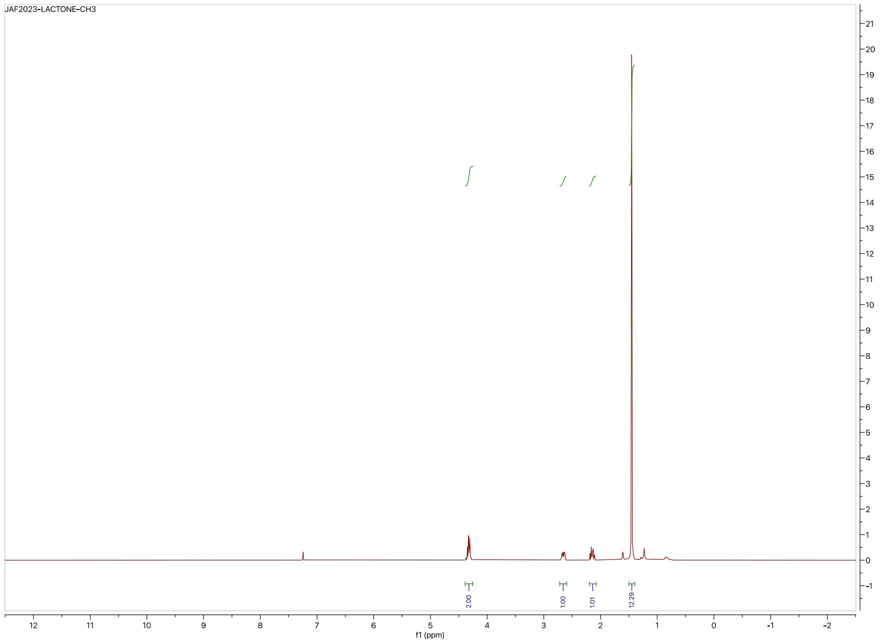


**Figure S21.** $^{1}$H NMR spectrum of **5a**.

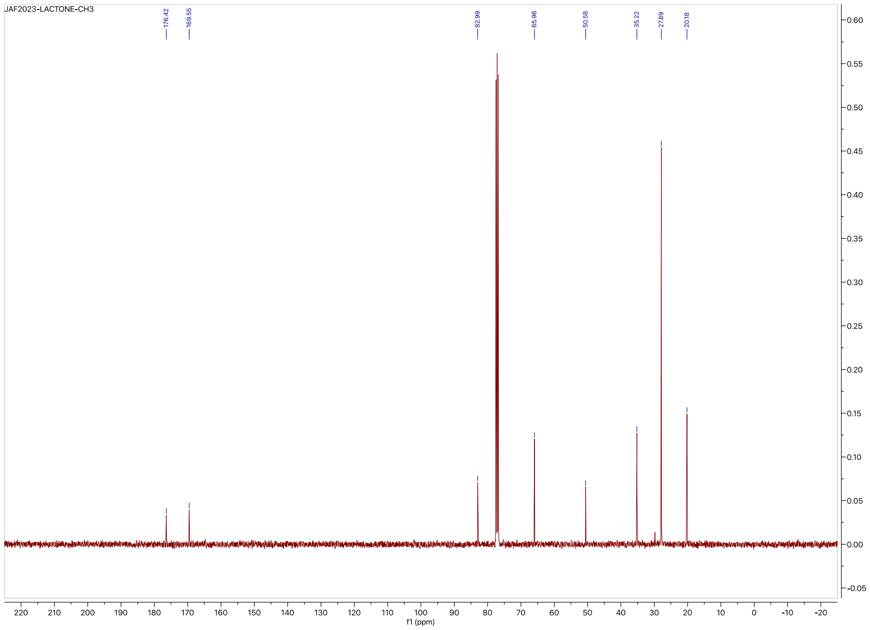


**Figure S22.** $^{13}$C NMR spectrum of **5a**.

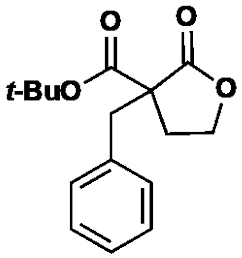


**5b**

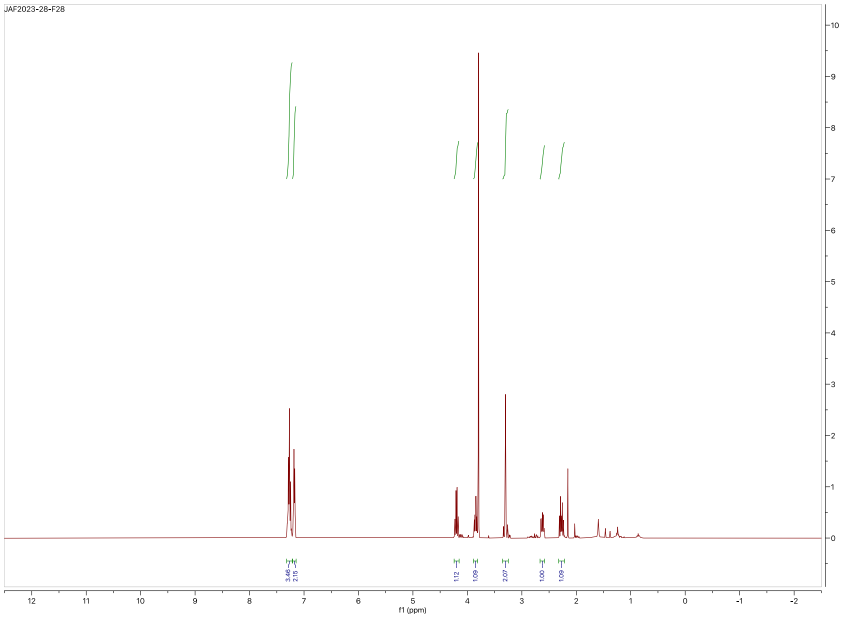


**Figure S23.** $^{1}$H NMR spectrum of **5b**.

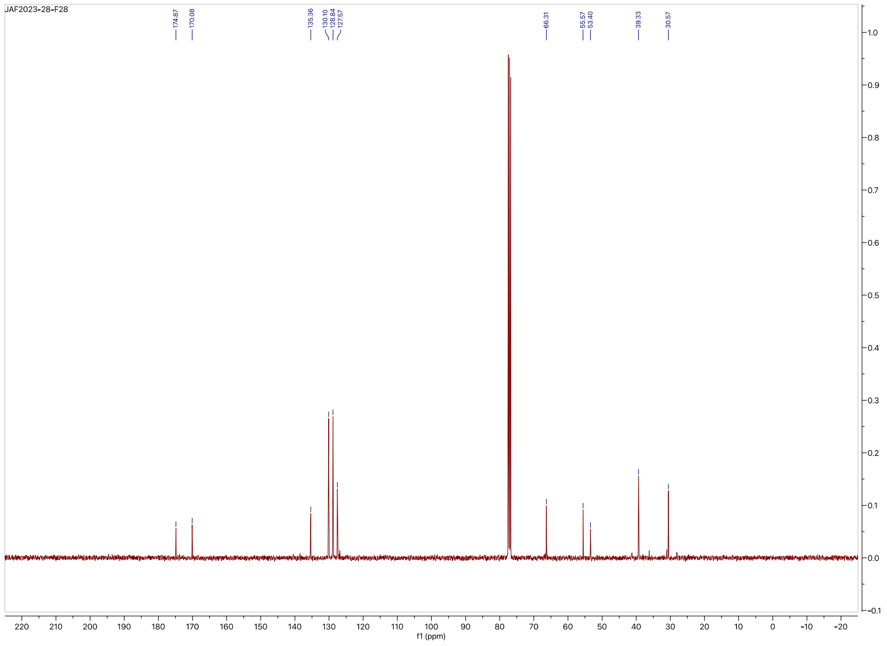


**Figure S24.** $^{13}$C NMR spectrum of **5b**.

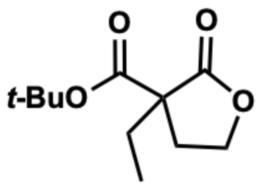

5c

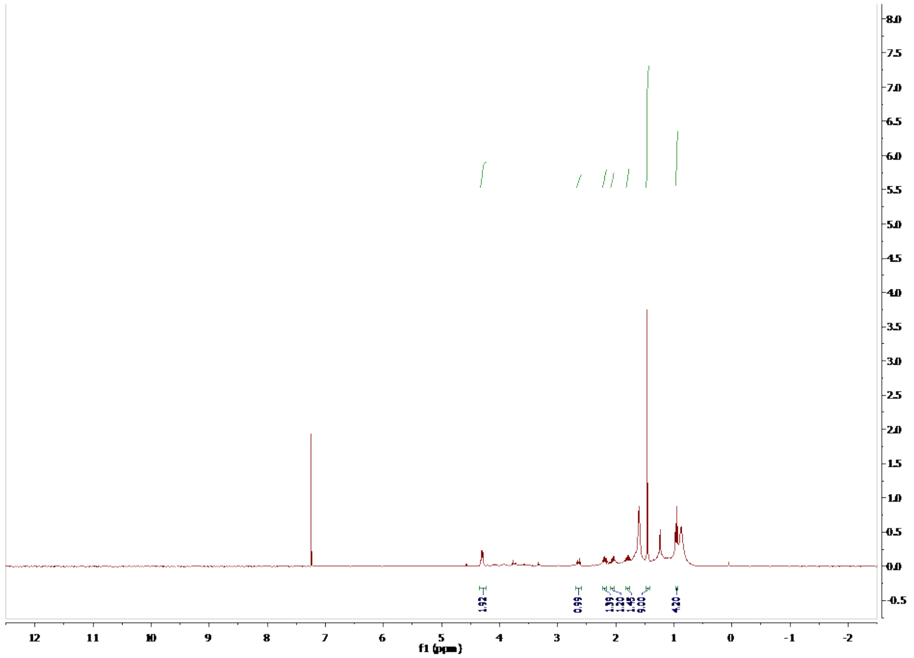

**Figure S25.** $^{1}$H NMR spectrum of **5c**.

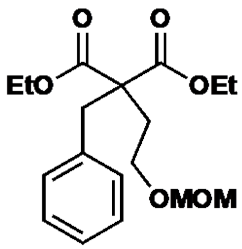


**7b**

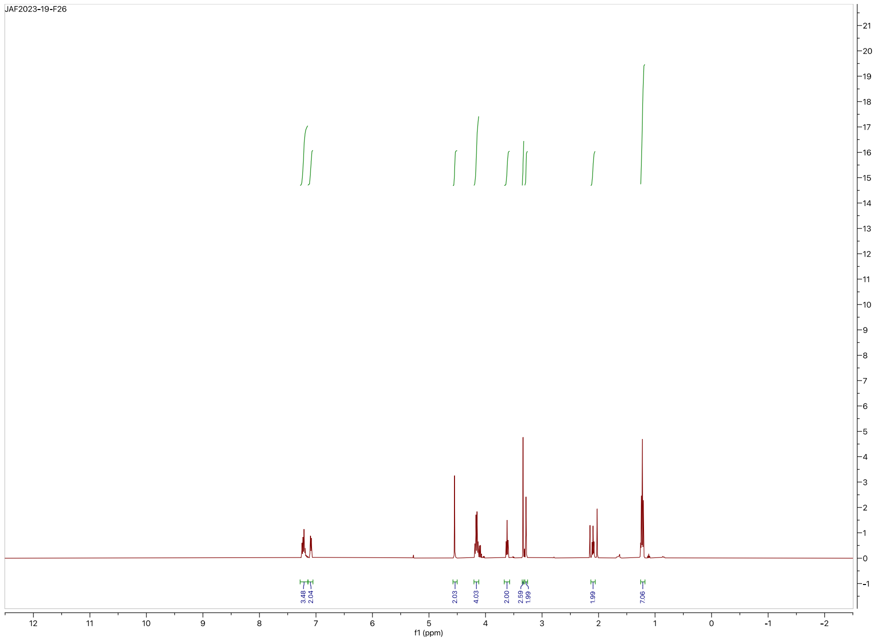


**Figure S26.** $^{1}$H NMR spectrum of **7b**.

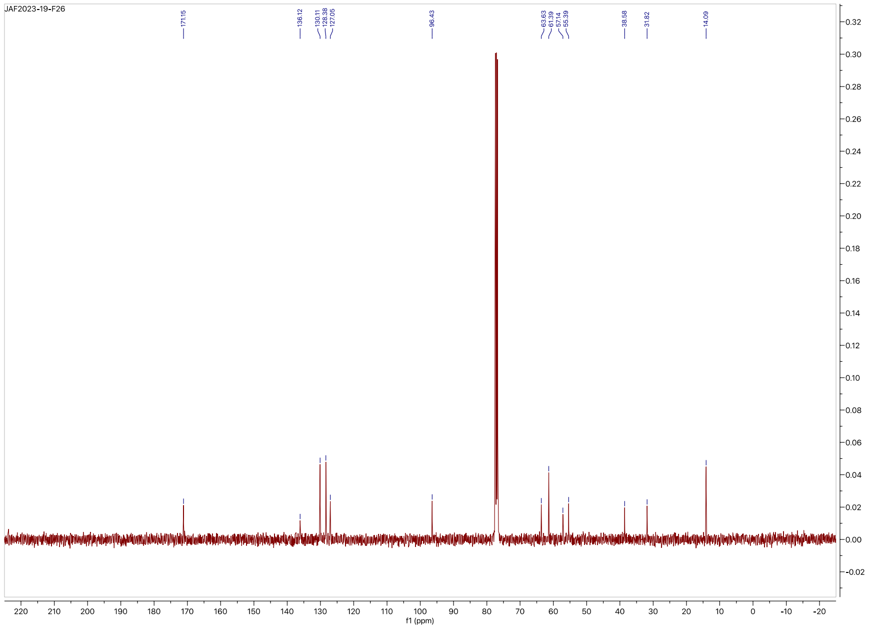


**Figure S27.** $^{13}$C NMR spectrum of **7b**.

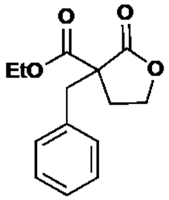


**8b**

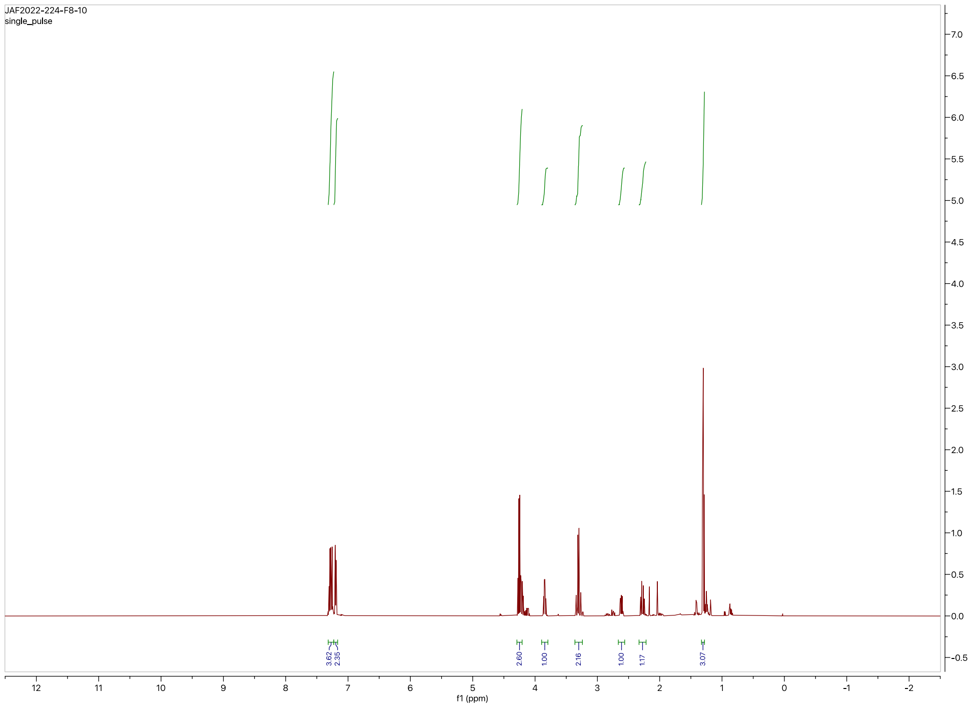


**Figure S28.** $^{1}$H NMR spectrum of **8b**.

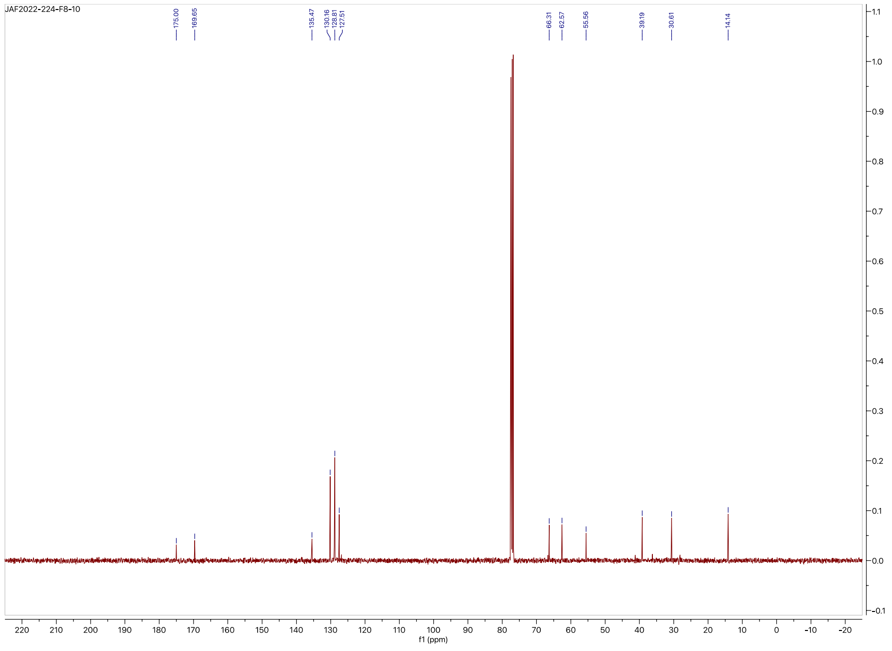


**Figure S29.** $^{13}$C NMR spectrum of **8b**.

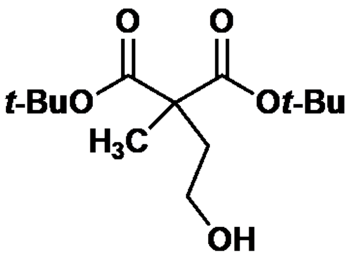

9a

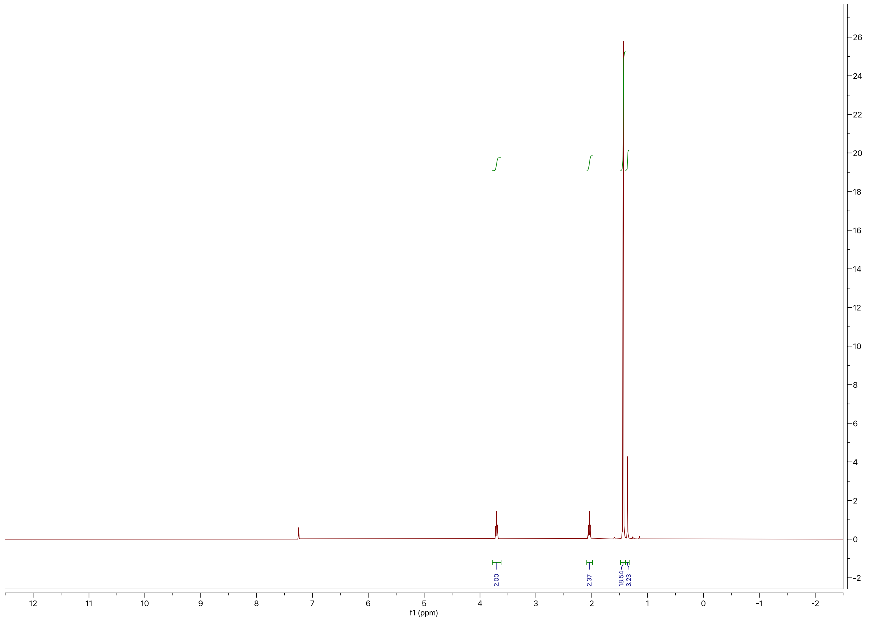

**Figure S30.** $^{1}$H NMR spectrum of **9a**.

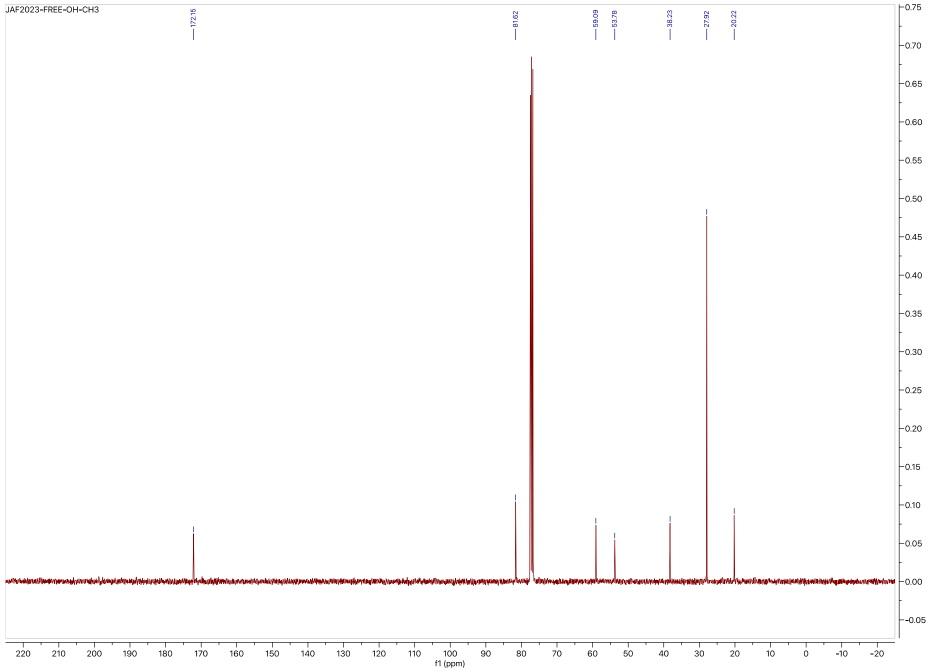

**Figure S31.** $^{13}$C NMR spectrum of **9a**.

## Copies of Chiral HPLC Chromatograms

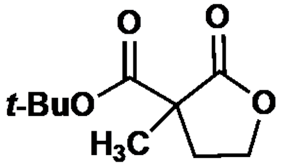


**5a**

Column: Chiralpak AD-H 4.6 mm x 250 mm x 5 µm; Eluent Rate: 1 mL/min; Eluent: 5% IPA/hexanes; Monitoring wave: 210 nm.

Racemic:

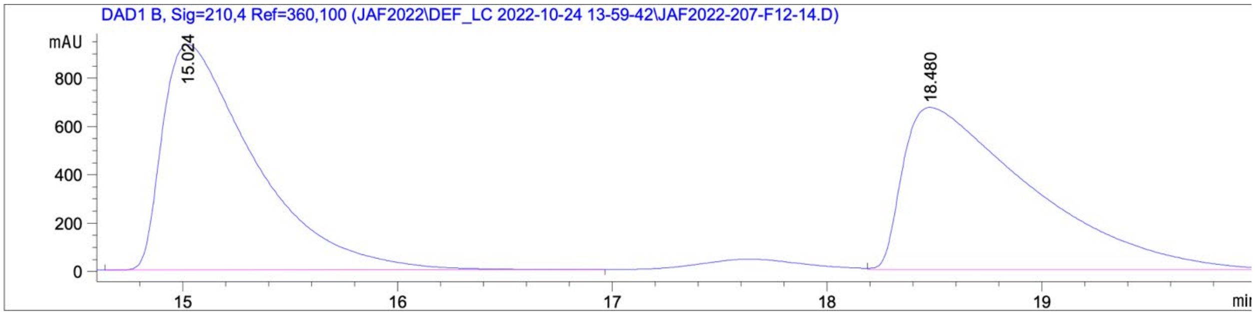


| Peak # | RetTime [min] | Sig | Type | Area [mAU*s] | Height [mAU] | Area % |
|---|---|---|---|---|---|---|
| 1 | 15.024 | 2 | BB | 2.77790e4 | 932.40704 | 47.4826 |
| 2 | 18.480 | 2 | VV | 2.79479e4 | 671.18896 | 47.7713 |

**Figure S32.** Racemic HPLC trace for compound **5a**.

Enantioenriched:

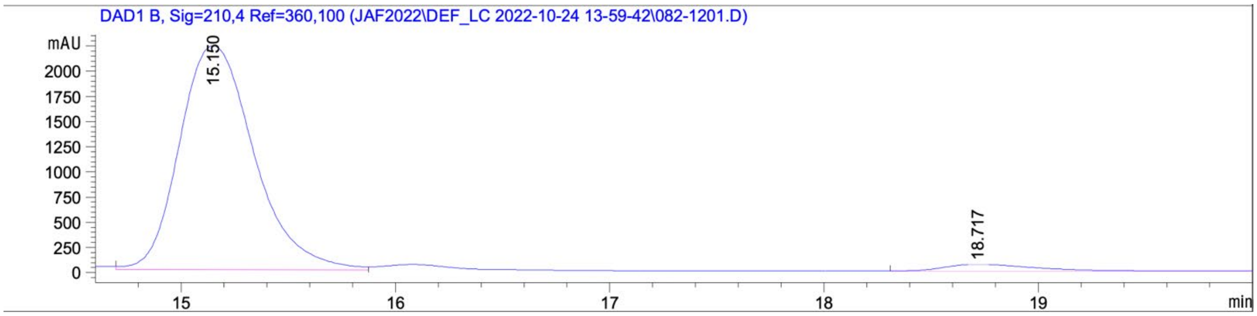


| Peak # | RetTime [min] | Sig | Type | Area [mAU*s] | Height [mAU] | Area % |
|---|---|---|---|---|---|---|
| 1 | 15.150 | 2 | VV | 5.38423e4 | 2225.26074 | 96.3389 |
| 2 | 18.717 | 2 | VB | 2048.12451 | 67.81987 | 3.6647 |

**Figure S33.** Enantioenriched HPLC trace for compound **5a**.

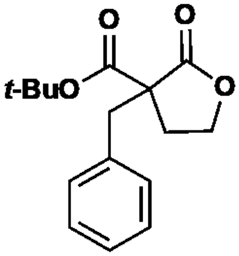


**5b**

Column: Chiralpak AD-H 4.6 mm x 250 mm x 5 µm; Eluent Rate: 1 mL/min; Eluent: 5% IPA/hexanes; Monitoring wave: 210 nm.
Racemic:

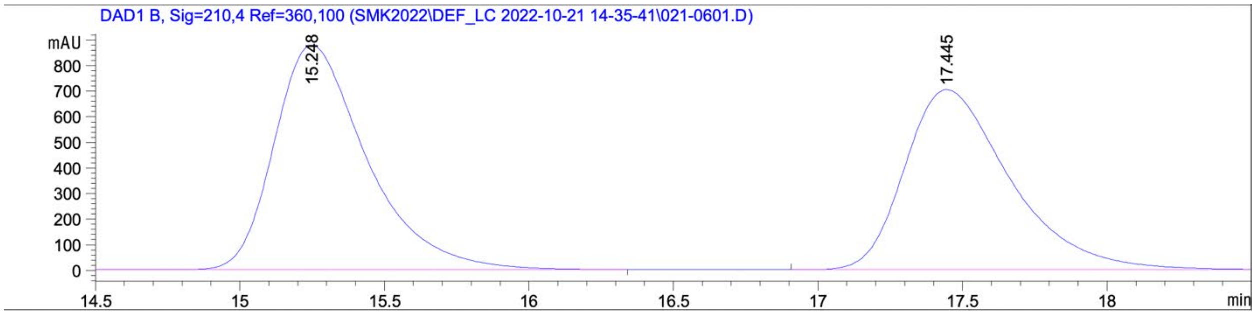


| Peak # | RetTime [min] | Sig | Type | Area [mAU*s] | Height [mAU] | Area % |
|---|---|---|---|---|---|---|
| 1 | 15.248 | 2 | BB | 1.95923e4 | 878.13434 | 49.4549 |
| 2 | 17.445 | 2 | BB | 1.78330e4 | 703.38556 | 45.0140 |

**Figure S34.** Racemic HPLC trace for compound **5b**.

Enantioenriched:

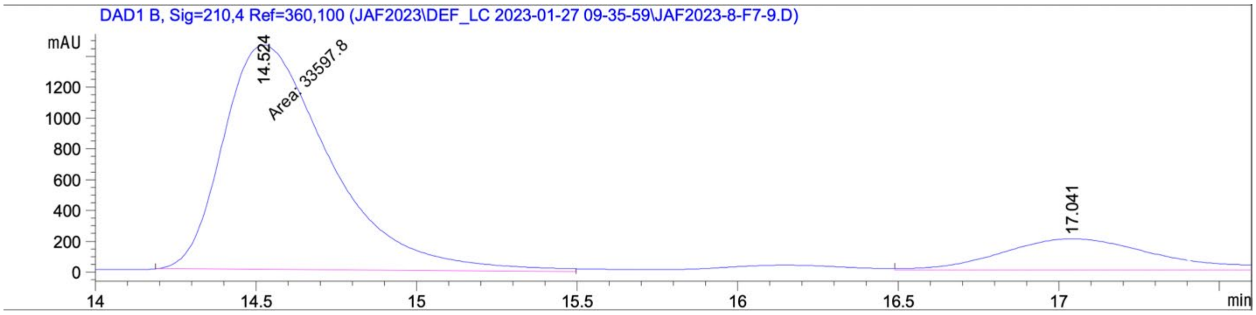


| Peak # | RetTime [min] | Sig | Type | Area [mAU*s] | Height [mAU] | Area % |
|---|---|---|---|---|---|---|
| 1 | 14.524 | 2 | MM | 3.35978e4 | 1453.78284 | 83.4784 |
| 2 | 17.041 | 2 | VV | 6651.47314 | 202.05029 | 16.5265 |

**Figure S35.** Enantioenriched HPLC trace for compound **5b**.

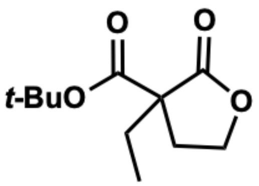

**5c**

Column: Chiralpak AD-H 4.6 mm x 250 mm x 5 µm; Eluent Rate: 1 mL/min; Eluent: 1% IPA/hexanes; Monitoring wave: 210 nm.

Racemic:

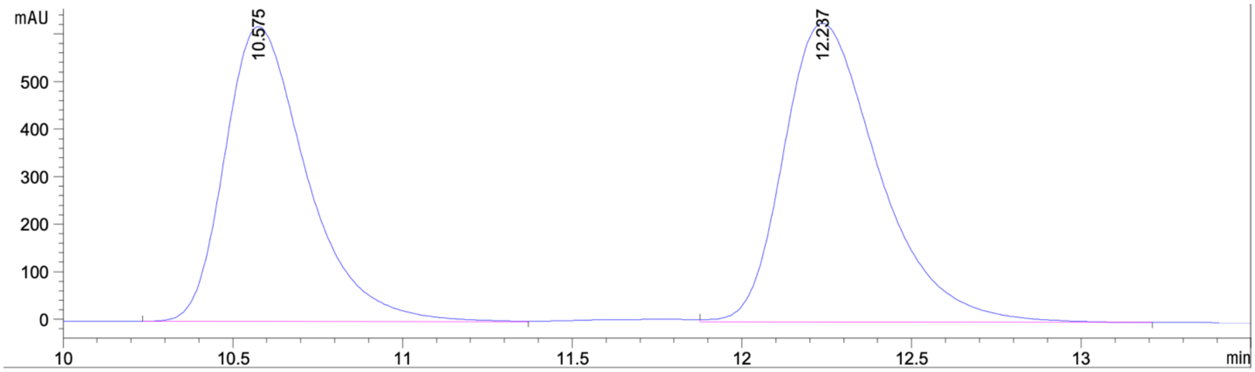


| Peak # | RetTime [min] | Type | Width [min] | Area [mAU*s] | Height [mAU] | Area % |
|---|---|---|---|---|---|---|
| 1 | 10.575 | BV | 0.2560 | 1.04079e4 | 619.55042 | 45.7817 |
| 2 | 12.237 | VV | 0.3030 | 1.23258e4 | 627.91461 | 54.2183 |

**Figure S36.** Racemic HPLC trace for compound **5c**.

Enantioenriched:

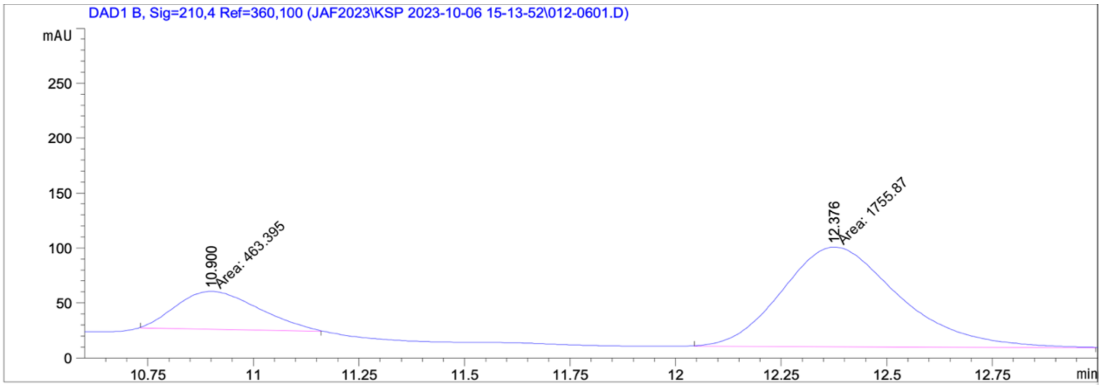


| Peak # | RetTime [min] | Sig | Type | Area [mAU*s] | Height [mAU] | Area % |
|---|---|---|---|---|---|---|
| 1 | 10.900 | 2 | MM | 463.39502 | 34.30749 | 20.8994 |
| 2 | 12.376 | 2 | MM | 1755.87048 | 90.67243 | 79.1908 |

**Figure S37.** Enantioenriched HPLC trace for compound **5c**.
